# Five New Alkaloids from the Stem Bark of *Daphniphyllum macropodum*

**DOI:** 10.3390/molecules19033055

**Published:** 2014-03-10

**Authors:** Yunyang Lu, Kai Gao, Xiaoyang Wang, Wei Zhang, Ning Ma, Haifeng Tang

**Affiliations:** 1 Department of Pharmacy, Xijing Hospital, Fourth Military Medical University, Xi’an 710032, China; E-Mails: luyunyanggq@163.com (Y.L.); gaokai19881220@163.com (K.G.); steveplum@sina.com (X.W.); zw2214146@163.com (W.Z.); maningtomoto@163.com (N.M.); 2 Institute of Materia Medica, School of Pharmacy, Fourth Military Medical University, Xi’an 710032, China

**Keywords:** *Daphniphyllum macropodum*, daphnicyclidins M and N, calyciphyllines Q–S, alkaloids, cytotoxicity

## Abstract

Five new alkaloids, daphnicyclidins M and N (compounds **1** and **2**) and calyciphyllines Q–S (compounds **3**–**5**), along with four known ones, paxiphylline C (**6**), macropodumine B (**7**), macropodumine C (**8**) and daphnicyclidin A (**9**) were isolated from the stem bark of *Daphniphyllum macropodum*. Calyciphylline Q (**3**) is the first calyciphylline A derivative possessing a double bond between C-18 and C-19. Their structures and relative configurations were elucidated on the basis of spectroscopic methods, especially 2D NMR techniques. Compounds **1**, **2**, **8** and **9** exhibited cytotoxic activity against P-388 cells with IC_50_ values of 5.7, 6.5, 10.3 and 13.8 µM, respectively. Compounds **1** and **2** also showed cytotoxic activity against SGC-7901 cells with IC_50_ values of 22.4 and 25.6 µM.

## 1. Introduction

Plants of genus *Daphniphyllum* are mainly distributed in southeast of Asia and are well known for producing highly polycyclic and structurally diverse alkaloids, which have drawn a great deal of attention from the biogenetic and synthetic points of view [1,2,3,4]. In recent years, a great number of *Daphniphyllum* alkaloids have been isolated and identified, and some of them exhibited significant cytotoxic activity against several human cancer cell lines [5,6,7,8,9,10,11,12,13,14,15,16,17,18,19].

**Figure 1:**
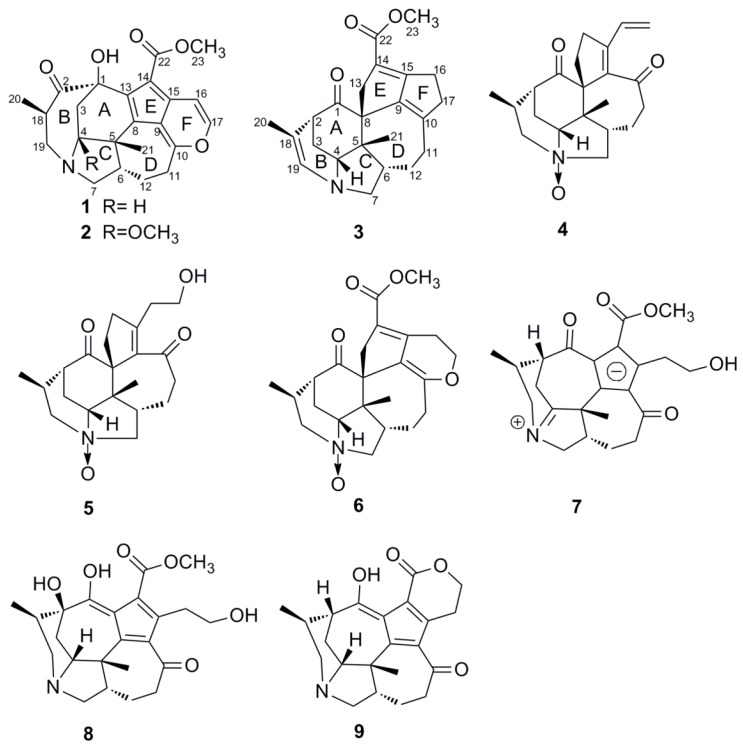
Structures of compounds **1**–**9**.

In the past years, a series of new bioactive compounds have been studied in our laboratory [20,21,22,23,24]. With the purpose of searching for bioactive and structurally unique *Daphniphyllum* alkaloids, an investigation of the extracts from the stem bark of *Daphniphyllum macropudum* was conducted, and this resulted in the isolation of five new alkaloids named daphnicyclidins M and N (compounds **1** and **2**) and calyciphyllines Q–S (compounds **3**–**5**), and four known related alkaloids **6**–**9** (Figure 1). More than 20 alkaloids have been isolated from the stem bark of *D. macropudum* and identified, including various structure types such as yuzurimine-type, daphnicyclidin-type, daphnezomine-type, calyciphylline-type, daphmanidin-type and daphniglaucin-type [25,26,27,28]. Compounds **1** and **2** are daphnicyclidin-type alkaloids, and **3**–**5** are calyciphylline-type alkaloids. The analog which shares a similar gross structure with daphnicyclidins M and N has been isolated for only once by Kobayashi [29]. Calyciphylline Q (**3**) is the first calyciphylline A derivative possessing a double bond between C-18 and C-19. This paper presents the isolation and structural elucidation of the new compounds **1**–**5**, along with their cytotoxic activities against four tumor cell lines, P-388 (mouse lymphocytic leukemia), A-549 (human lung carcinoma), SGC-7901 (human gastric carcinoma) and HL-60 (human promyelocytic leukemia).

## 2. Results and Discussion

Daphnicyclidin M (**1**) was obtained as light yellow powder. The molecular formula was determined as C_23_H_25_NO_5_ by HREIMS at *m/z* 418.1632 ([M+Na]^+^, calcd for C_23_H_25_NO_5_Na, 418.1630), which indicated 12 degrees of unsaturation. ^13^C-NMR (Table 1) and DEPT spectra revealed 23 carbon signals due to three tetrasubstituted olefins, one disubstituted olefin, two carbonyls, two sp^3^ quaternary carbons, three sp^3^ methines, five sp^3^ methylenes, two sp^3^ methyls and one methoxy group. Among them, two methylenes (δ_C_ = 60.1, δ_H_ = 2.37 and 3.07; δ_C_ = 53.1, δ_H_ = 2.63 and 3.06) and one methine (δ_C_ = 67.9, δ_H_ = 3.50) were ascribed to those bearing a nitrogen, while two olefin carbons (δ_C_ = 168.4 and δ_C_ = 146.1, δ_H_ = 7.93) and one sp^3^ quaternary carbon (δ_C_ = 77.8) were assigned to those bearing oxygen atoms. Since six out of 12 degrees of unsaturation were accounted for, **1** was inferred to possess six rings.

**Table 1:** ^1^H-NMR (500 MHz) and ^13^C-NMR (125 MHz) data for compounds **1**–**5** (δ in ppm, *J* in Hz).

C	1 ^a^		2 ^a^		3 ^a^		4 ^a^		5 ^b^	
	δ_C_	δ_H_	δ_C_	δ_H_	δ_C_	δ_H_	δ_C_	δ_H_	δ_C_	δ_H_
1	77.8	-	78.9	-	209.2	-	217.6	-	216.4	-
2	213.1	-	212.4	-	44.4	2.93 (t, 3.1)	43.3	2.38 (brd, 5.0)	41.5	2.28–2.31 (m)
3a 3b	31.4	2.26 (d, 3.4) 2.26 (d, 3.4)	33.9	2.18 (d, 14.8) 2.35 (d, 14.8)	24.4	1.97 (dt, 14.1, 2.7) 2.26 (dt, 14.2, 3.1)	34.6	1.86–1.92 (m) 2.76–2.78 (m)	18.6	2.38–2.40 (m) 2.64–2.66 (m)
4	67.9	3.50 (t, 3.3)	98.0	-	64.6	3.07–3.09 (m)	90.4	4.00 (t, 2.2)	87.9	3.93 (br s)
5	46.8	-	53.3	-	55.9	-	54.7	-	52.5	-
6	46.7	2.56–2.58 (m)	42.3	2.85–2.88 (m)	58.0	1.78–1.85 (m)	47.2	3.01–3.07 ^c^	45.2	2.89 (t, 7.6)
7a 7b	60.1	2.37 (t, 9.6) 3.06–3.08 (m)	59.4	2.31 (dd, 8.2, 2.8) 3.13–3.16 (m)	61.9	2.92 (d, 9.4) 3.28–3.31 (m)	69.6	3.12 (t, 6.3) 3.51 (t, 13.2)	67.4	3.04–3.09 (m) 3.26–3.32 (m)
8	126.7	-	128.5	-	57.3	-	72.3	-	69.7	-
9	108.8	-	108.2	-	150.7	-	139.7	-	136.9	-
10	168.4	-	168.1	-	151.7	-	206.1	-	202.8	-
11a 11b	31.0	2.98–3.01 (m) 3.67–3.73 (m)	31.1	2.94–2.98 (m) 3.65–3.69 (m)	34.5	2.06–2.14 (m) 2.36 (d, 18.5)	37.2	2.32–2.35 (m) 2.32–2.35 (m)	36.0	2.12–2.15 (m) 2.12–2.15 (m)
12a 12b	28.2	1.69–1.73 (m) 2.57–2.59 (m)	27.6	1.67–1.75 (m) 2.54–2.58 (m)	32.1	1.32–1.37 (m) 1.78–1.85 (m)	19.4	1.86–1.92 (m) 2.01–2.08 (m)	17.8	1.71–1.74 (m) 1.91–1.98 (m)
13a 13b	142.0	- -	141.5	- -	47.6	2.81 (d, 16.5) 3.40 (d, 17.7)	20.2	2.44–2.50 (m) 2.78–2.82 (m)	32.9	1.68–1.71 (m) 2.62–2.65 (m)
14a 14b	123.2	- -	123.3	- -	115.9	- -	33.2	2.64 (t, 8.7) 2.74–2.77 (m)	35.9	2.32–2.37 (m) 2.50–2.52 (m)
15	134.4	-	134.7	-	173.5	-	154.6	-	156.4	-
16a 16b	111.8	7.70 (d, 5.3) -	117.7	7.69 (d, 5.3) -	26.8	2.74–2.78 (m) 2.74–2.74 (m)	132.5	6.89 (dd, 10.8, 6.7) -	33.3	2.18–2.23 (m) 2.66–2.70 (m)
17a 17b	146.1	7.93 (d, 5.3) -	145.9	7.92 (d, 5.3) -	41.7	2.78–2.80 (m) 2.85–2.87 (m)	122.2	5.42 (d, 10.8) 5.52 (d, 17.5)	59.1	3.43–3.45 (m) 3.50–3.53 (m)
18	36.9	2.66–2.70 (m)	39.1	2.49–2.53(m)	112.8	-	33.0	2.57–2.62(m)	31.0	2.43–2.47 (m)
19a 19b	53.1	2.61–2.65 (m) 3.02–3.06 (m)	53.8	2.91 (dd, 15.6, 2.8) 3.17 (dd, 15.2, 2.3)	135.8	5.77(s) -	68.2	3.01–3.07 ^c^ 3.63 (dd, 13.3, 7.1)	66.1	2.99–3.04 (m) 3.55–3.57 (m)
20	14.0	0.81 (3H, d, 6.7)	13.3	0.83 (3H, d, 6.7)	19.9	1.70 (3H, s)	19.6	1.14 (3H, d, 6.7)	18.9	1.03 (3H, d, 6.7)
21	28.8	1.48 (3H, s)	25.6	1.46 (3H, s)	27.2	1.20 (3H, s)	23.2	1.50 (3H, s)	22.1	1.39 (3H, s)
22	169.0	-	169.0	-	168.1	-	-	-	-	-
23	51.9	3.83 (3H, s)	51.8	3.82 (3H, s)	51.8	3.70 (3H, s)	-	-	-	-
4-OMe	-	-	50.0	3.35 (3H, s)	-	-	-	-	-	-

^a^ Measured in CD_3_OD; ^b^ Measured in DMSO-*d_6_*; ^c^ Overlapped.

**Figure 2:**
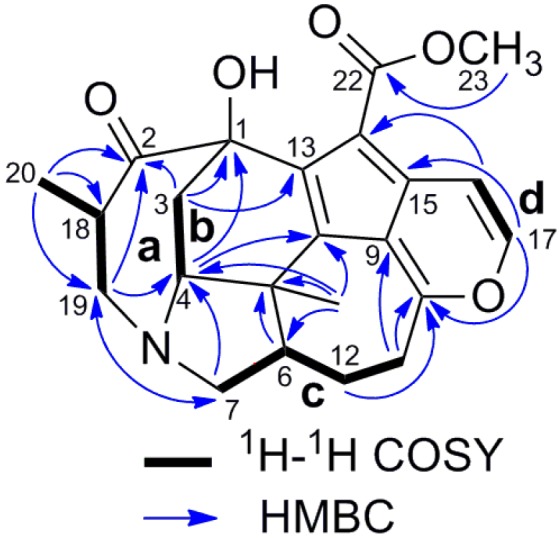
Selected 2D NMR correlations of daphnicyclidin M (**1**).

**Figure 3:**
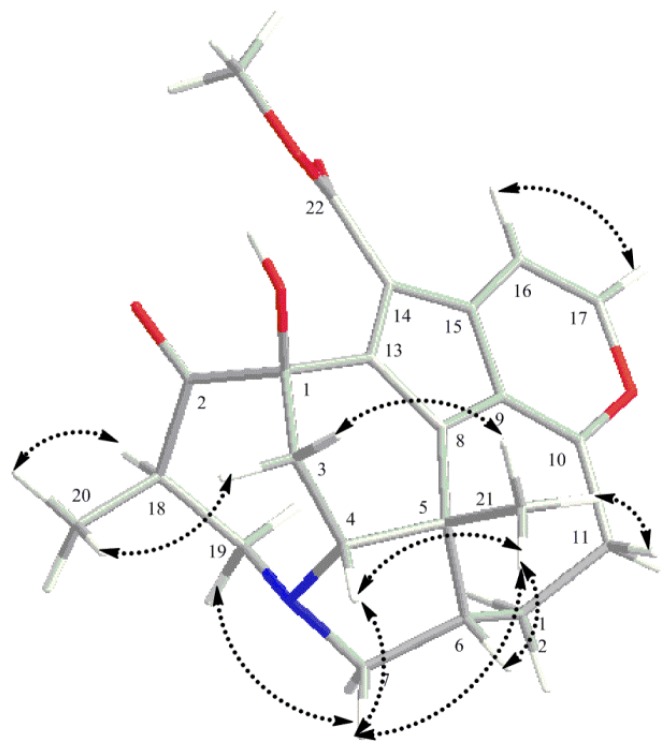
Key NOESY correlations of daphnicyclidin M (**1**).

Four partial structures: **a** (C-18 to C-19 and C-20), **b** (C-3 to C-4), **c** (C-6 to C-7 and C-12, and C-11 to C-12) and **d** (C-16 to C-17) were deduced from the extensive analysis of the 2D NMR data of **1**, including HSQC, ^1^H-^1^H COSY and HMBC, as shown in Figure 2. The HMBC correlations from H_2_-3 to C-1, C-2 and C-13, and H-4 to C-1 indicated C-2, C-3 and C-13 were all connected to C-1; H-19b and H_3_-20 to C-2 suggested the connectivity between C-18 and C-2. HMBC correlations from H-7b to C-4, H-7a to C-19 and H-19a to C-4 and C-7 gave rise to the connectivity of partial structures **a**, **b** and **c** through a nitrogen atom. The connections between C-4, C-6 and C-8 to C-21 through C-5 were confirmed by the HMBC correlations from H_2_-3 and H-4 to C-5, H-4 and H-6 to C-8 and H_3_-21 to C-4, C-5, C-6 and C-8, and this constructed the ring C. HMBC correlations from H_2_-12 and H_2_-11 to C-10 and H-11b to C-9 implied that C-11 and C-9 were connected through C-10. The presence of ring F was elucidated by the chemical shifts of C-10 (δ_C_ = 168.4) and C-17 (δ_C_ = 146.1), and HMBC correlations of H-17 to C-10 and C-15, and H-16 to C-9. The ring E and the methoxy carbonyl group at C-14 were deduced from a comprehensive analysis of the chemical shifts [126.7 (C-8), 108.8 (C-9), 168.4 (C-10), 142.0 (C-13), 123.2 (C-14), 134.4 (C-15)] and HMBC correlation of H-16 to C-14, and H_3_-23 to C-22. The relative configuration of **1** was elucidated by NOESY correlations as depicted in a computer-generated three-dimensional drawing, as shown in Figure 3.

The NOESY correlations of H_3_-21 to H-4 and H-4 to H-6 indicated that H-4 and H-6 were in the β-orientation. The α-orientation of H-18 was deduced from the NOESY correlations of H-18 to H-7a and H-7b to H-4. Thus, it was clear that the ring B took a boat conformation. In consideration of biosynthetic pathway [20] and the boat conformation of ring B, the OH group at C-1 must be β-oriented. The NOESY correlations of H_3_-21 to H-11b implied that ring D took a twist-boat conformation, similar to that of daphnicyclidin A [30]. Thus, the structure of daphnicyclidin M was assigned as **1**, which is the C-4 dehydroxylated, C-16 and C-17 dehydrogenated derivative of daphnicyclidin K [29].

Daphnicyclidin N (**2**) showed a molecular formula of C_24_H_27_NO_6_, as determined by HREIMS at *m/z* 448.1738 ([M+Na]^+^, calcd. for C_24_H_27_NO_6_Na, 448.1736). The comparison of the ^1^H-NMR and ^13^C-NMR (Table 1) data of **2** with those of **1** suggested that the two alkaloids shared the same gross structure. The main difference bteween the two alkaloids was the fact that the molecular weight of **2** was larger than that of **1** by 30 units. Thus, it was proposed that the H-4 was replaced by a methoxy group. This was proved by the chemical shift of C-4 (δ_C_ = 98.0) which was shifted downfield ∆δ_C_ = +30.1 as compared with that of **1**, and the HMBC cross-peak of the H_3_ signal (δ_H_ = 3.35, s) to C-4 (Supporting Information). The relative configuration of **2** was the same as that of **1**, thus, OH-1, H-6, CH_3_-20 and CH_3_-21 were also β-oriented. Because the chemical shift of C-21 (δ_C_ = 25.6) was shifted upfield (∆δ_C_ = −2.65) for the γ-steric compression effect from oxygen atom of C-4, the methoxy group at C-4 was also deduced as the β-orientation [31].

Calyciphylline Q (**3**) was obtained as a light yellow oil, exhibiting a pseudomolecular ion peak at *m/z* 388 [M+Na]^+^ in the ESIMS. The molecular formula C_23_H_27_NO_3_ of **3** was established by HRESIMS at *m/z* 388.1890 ([M+Na]^+^, calcd. for C_23_H_27_NO_3_Na, 388.1889), corresponding to 11 degrees of unsaturation. The ^13^C-NMR (Table 1) and DEPT spectra showed 23 carbon signals including two carbonyls, three double bonds, two sp^3^ quaternary carbons, three sp^3^ methines, seven sp^3^ methylenes, two sp^3^ methyls and one methoxy group. Among them one methylene (δ_C_ = 61.9, δ_H_ = 2.92 and 3.30), one methine (δ_C_ = 64.6, δ_H_ = 3.09) and one double bond carbon (δ_C_ = 135.8, δ_H_ = 5.77) were assigned to those bearing a nitrogen.

**Figure 4:**
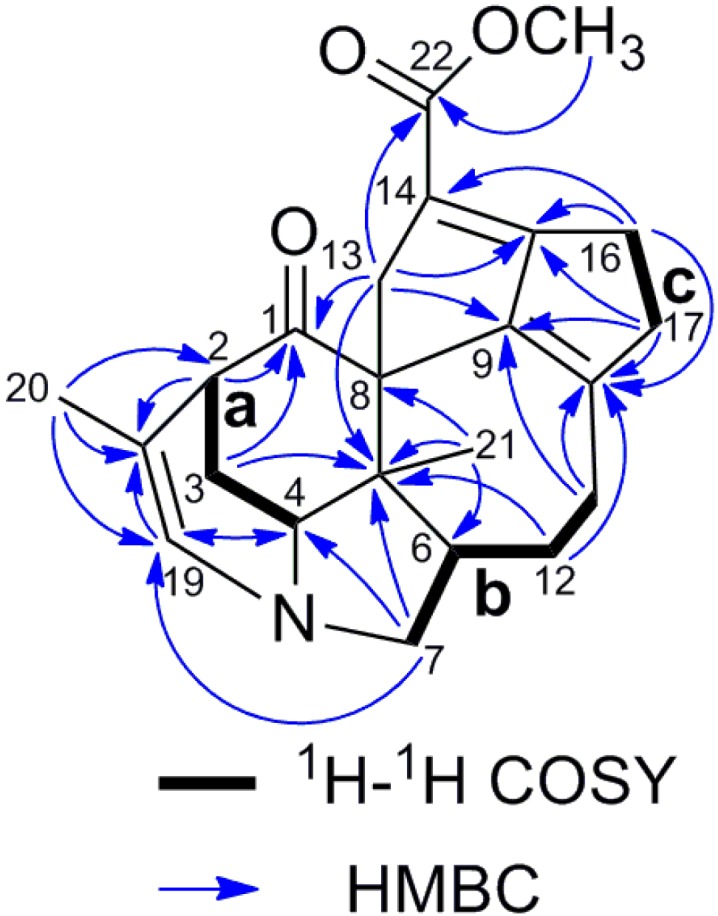
Selected 2D NMR correlations of calyciphylline Q (**3**).

**Figure 5:**
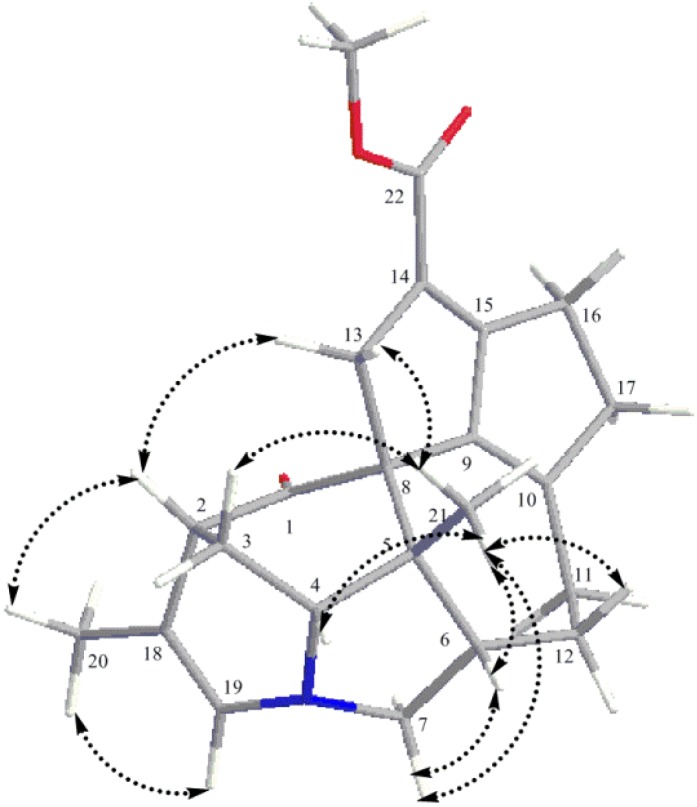
Key NOESY correlations of calyciphylline Q (**3**).

The ^1^H-^1^H COSY spectrum of **3** revealed the connectivities of three structure fragments **a** (C-2 to C-4), **b** (C-6 to C7 and C-12, and C-11 to C-12) and **c** (C-16 to C-17) as shown in Figure 4. The connection of C-4, C-7 and C-19 to each other through a nitrogen atom was deduced from the HMBC correlations from H-4 to C-19, H-7a to C-4, and H-7b to C-19 and H-19 to C-4. The ring B was elucidated by the HMBC correlations of H-2 to C-18, H-19 to C-2, C-18 and C-20, H_3_-20 to C-2, C-18 and C-19. A ketone carbonyl at C-1 was revealed from HMBC cross-peaks of H-2 and H-3a to C-1. The HMBC correlations from H_3_-21 to C-4, C-5 and C-6, from H-3a, H-7a and H-12a to C-5 indicated the connectivities of C-21 to C-4 and C-6 via C-5. The connectivity of fragment **b** and **c** through C-10 and the presence of ring F were suggested by the HMBC correlations from H-11a, H_2_-16 and H-17a to C-10, from H_2_-11 and H-17b to C-9, and from H_2_-16 and H-17a to C-15. The linkages of C-13 to C-1, C-5 and C-9 through C-8 were confirmed by the HMBC correlations of H_2_-13 to C-5, H_3_-21 to C-5 and C-8, and H-13a to C-1 and C-9. The ring E was elucidated on the basis of the HMBC correlations of H-13a to C-15, H_2_-16 to C-15 and C-14 and H-17a to C-15. The methoxycarbonyl group at C-14 was deduced from a comprehensive analysis of the chemical shifts [150.7 (C-9), 151.7 (C-10), 115.9 (C-14), 173.5 (C-15)] and HMBC correlation of H-13b to C-22, H_2_-16 to C-14, and H_3_-23 to C-22. The relative configuration of **3** was elucidated by NOESY spectrum as shown in Figure 5. The NOESY correlations of H_3_-21 to H-3b, H-4 and H-6 indicated that H-3b, H-4, H-6 and CH_3_-21 were all on the same side, and assumed to be in β-orientation just the same as those of daphniyunnine A [32]. The β-orientation of H-2 was implied by the NOESY correlation of H-2 with H-13a. The correlation of H-13b to H_3_-21 suggested that C-13 was β-oriented. The boat conformation of ring D was deduced from the NOESY correlation of H_3_-21 to H-12a. Thus, the structure of calyciphylline Q was elucidated as **3**.

Calyciphylline R (**4**) exhibited a pseudomolecular ion peak at *m/z* 342 [M+H]^+^ in the ESIMS, and the molecular formula was established as C_21_H_27_NO_3_ by HRESIMS at *m/z* 342.2068 ([M+H]^+^, calcd. for C_21_H_28_NO_3_, 342.2069), corresponding to nine degrees of unsaturation. The ^13^C-NMR (Table 1) of **4** revealed 21 carbon resonances, which were classified into two carbonyls, two double bonds, two sp^3^ quaternary carbons, four sp^3^ methines, seven sp^3^ methylenes, and two methyl groups. One methine (δ_C_ = 90.4, δ_H_ = 4.00) and two methylenes (δ_C_ = 68.2, δ_H_ = 3.04 and 3.63; δ_C_ = 69.6, δ_H_ = 3.12 and 3.51) were ascribed to those bearing an oxidative nitrogen. Comparison of the NMR (^1^H-NMR, ^13^C-NMR, HSQC, ^1^H-^1^H COSY and HMBC) spectra of **4** with those of daphlongamine E [33], suggested that the two compounds are closely related. However, significant downfield changes of the chemical shifts of C-4 (δ_C_ = 90.4), C-7 (δ_C_ = 69.6) and C-19 (δ_C_ = 68.2) in relation to those of daphlongamine E (C-4 (δ_C_ = 65.5), C-7 (δ_C_ = 53.6) and C-19 (δ_C_ = 49.8)) indicated that the former one is the N-oxide form of the latter one [34]. Thus, the relative configuration of **4** is the same as daphlongamine E.

Calyciphylline S (**5**) showed a pseudomolecular ion peak at *m/z* 360 [M+H]^+^ in the ESIMS, and the molecular formula was determined as C_21_H_29_NO_4_ by HRESIMS at *m/z* 360.2172 ([M+H]^+^, calcd. for C_21_H_30_NO_4_, 360.2175), with 8 degrees of unsaturation. The ^13^C-NMR (Table 1) and DEPT spectra of **5** revealed 21 carbon signals, ascribed to two carbonyls, one tetrasubstituted olefin, two sp^3^ quaternary carbons, four sp^3^ methines, nine sp^3^ methylenes and two sp^3^ methyl groups. Among them, one methine (δ_C_ = 87.9, δ_H_ =3.93) and two methylenes (δ_C_ = 66.1, δ_H_ = 3.04 and 3.56; δ_C_ = 67.4, δ_H_ = 3.10 and 3.30) were ascribed to those bearing an oxidative nitrogen. Since three out of eight degrees of unsaturation have been accounted for, **5** was inferred to possess five rings. A comparison of the ^13^C chemical shifts of C-4 (δ_C_ = 87.9), C-7 (δ_C_ = 67.4) and C-19 (δ_C_ = 66.1) in **5** with those of daphniyunnine B (longeracinphyllin B) indicated the presence of an N-oxide group attached to those C-atoms [32,35]. Thus calyciphylline S was inferred to be the N-oxide form of daphniyunnine B (longeracinphyllin B), which was confirmed by the 2D NMR (HSQC, ^1^H-^1^H COSY and HMBC) spectra of **5** (Supporting Information). The structure of calyciphylline S (**5**) could also be deduced from the comparison of the ^1^H-NMR and ^13^C-NMR data (Table 1) of **5** with those of **4**. It could be easily inferred that compound **5** was the water addition product of **4** at C-16 and C-17, since one methine (δ_C_ = 132.5, δ_H_ = 6.89) and one methylene (δ_C_ = 122.2, δ_H_ = 5.42 and 5.52) disappeared in compound **4** while two methylenes (δ_C_ = 33.3, δ_H_ = 2.22 and 2.68; δ_C_ = 59.1, δ_H_ = 3.44 and 3.51) emerged in **5**.

Four known alkaloids were identified as paxiphylline C [36], macropodumine B [37], macropodumine C [37] and daphnicyclidin A [30] on the basis of the comparison of their ^1^H-NMR, ^13^C-NMR and ESIMS data with that reported.

The cytotoxicity of the new compounds were evaluated against mouse lymphocytic leukemia P-388 cells, human lung carcinoma A-549 cells, human gastric carcinoma SGC-7901 cells and human promyelocytic leukemia HL-60 cells using the 3-(4,5-dimethylthiazol-2-yl)-2,5-diphenyltetrazolium bromide (MTT) colorimetric assay method *in vitro* [38]. As shown in Table 2, compounds **1**, **2**, **8** and **9** exhibited cytotoxic activity against P-388 cells with IC_50_ values of 5.7, 6.5, 10.3 and 13.8 µM, respectively. Compounds **1** and **2** also showed a moderate cytotoxic activity against SGC-7901 cells with IC_50_ values of 22.4 and 25.6 µM. Compounds **3**, **4**, **5**, **6** and **7** were inactive (IC_50_ > 50 µM) against to the cell lines above.

**Table 2:** Cytotoxic activity of compounds **1**–**9** against four cancer cell lines *in vitro*.

Compounds ^a^	Cytotoxic activity (IC_50_, µM)			
	P-388	A-549	SGC-7901	HL-60
**1**	5.7	>50	22.4	>50
**2**	6.5	>50	25.6	>50
**8**	10.3	>50	>50	>50
**9**	13.8	>50	>50	>50
Cisplatin ^b^	0.3	0.9	3.2	1.1

P-388 = mouse lymphocytic leukemia cell line; A-549 = human lung carcinoma cell line; SGC-7901 = human gastric carcinoma cell line; HL-60 = human promyelocytic leukemia cell line; ^a^ Compounds **3**, **4**, **5**, **6** and **7** were inactive (IC_50_ > 50 µM) against all cell lines; ^b^ Cisplatin was used as positive control.

## 3. Experimental

### 3.1. General Information

Optical rotations were measured with a Perkin-Elmer 343 polarimeter. 1D and 2D NMR spectra were recorded on a Bruker AVANCE-500 spectrometer with TMS as internal standard. ESIMS and HRESIMS were carried out on a Micromass Quattro mass spectrometer. HPLC was carried out on a Dionex P680 liquid chromatograph equipped with a UV 170 UV/Vis detector using a YMC-Pack R&D ODS A column (20 × 250 mm i.d., 5 μm, YMC Co., Ltd., Kyoto, Japan) and monitored at 225, 250, 275, 300 nm, simultaneously. Column chromatographies were performed on silica gel (200–300 mesh and 300–400 mesh; Qingdao Marine Chemical Inc., Qingdao, P. R. China), reversed phase silica gel (Lichroprep RP-18, 40–63 µm, Merck Inc., New York, NY, USA), and Sephadex LH-20 (40–70 µm, GE-Healthcare, Uppsala, Sweden). Chemical reagents for isolation were of analytical grade and purchased from Tianjin Fuyu Chemical Co. Ltd. (Tianjin, China).

### 3.2. Plant Material

The stem bark of *Daphniphyllum macropodum* was collected in Chongqing Province, People’s Republic of China, in November 2012, and identified by associate researcher Maoxiang Lin of the Chongqing Institute of Medicinal Plant Cultivation. A voucher specimen (XJ-T20121215) was deposited in the Herbarium of the Department of Pharmacy, Xijing Hospital, Fourth Military Medical University.

### 3.3. Extraction and Isolation

The air-dried and powdered stem bark (20.0 kg) of *Daphniphyllum macropodum* was extracted three times with refluxing 95% EtOH (200 L, 2 h each time). After removal of the solvent under reduced pressure, the extract (2.1 kg) was dispersed in water and adjusted with 1% HCl to pH 2–3, then filtered. The aqueous phase was adjusted to pH 10 with 2 mol∙L^−1^ NaOH followed by extraction with CHCl_3_ to get the crude alkaloid (45.0 g). The crude alkaloid was subjected to a silica gel column eluting with a CHCl_3_/CH_3_OH (1:0 to 0:1) gradient to obtain four major fractions (A to D). Fraction A (15.5 g) was chromatographed on a silica gel column eluting with a CHCl_3_/CH_3_OH (20:1 to 10:1) gradient to give ten further fractions (A1–A10). Fraction A5 was subjected to size exclusion chromatography on a Sephadex LH-20 column equilibrated with CH_3_OH to remove the pigments and impurities, then was further purified by HPLC to afford compounds **1** (7.7 mg, *t*_R_ = 20.2 min), **2** (6.2 mg, *t*_R_ = 21.2 min) and **4** (5.5 mg, *t*_R_ = 33.6 min) eluting with MeOH/H_2_O (8:3) at a flow rate of 6 mL/min. Paxiphylline C (5.7 mg, *t*_R_ = 18.1 min) and compound **3** (4.4 mg, *t*_R_ = 24.5 min) were obtained from fraction A7 by HPLC eluting with MeOH/H_2_O (7:3) at a flow rate of 8 mL/min. Fraction A10 was purified by HPLC to give compound **5** (7.2 mg, *t*_R_ = 22.5 min) eluting with MeOH/H_2_O (6:4) at a flow rate of 6 mL/min. Fraction B (15.0 g) was subjected to silica gel column chromatography eluting with a CHCl_3_/CH_3_OH (10:1 to 0:1) gradient to afford two fractions (B1 and B2). Fraction B1 was purified over a Sephadex LH-20 column equilibrated with CH_3_OH to yield macropodumine C (15.8 mg). Fraction C (3.4 g) was chromatographed over a Sephadex LH-20 column equilibrated with CH_3_OH to remove the pigments and impurities, and finally purified by means of HPLC eluting with MeOH/H_2_O (4:6) to yield daphnicyclidin A (4.8 mg) in 27.5 min and macropodumine B (17.6 mg) in 37.6 min.

*Daphnicyclidin M* (**1**): amorphous light yellow powder; 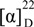 −40.3 (*c* 0.11, MeOH); UV (MeOH) λ_max_ (log ε) 274 (1.36), 221 (1.26) nm; IR (KBr) *ν*_max_ 3423, 2915, 1690, 1643, 1616, 1522, 1454, 1375, 1127, 984 cm^−1^; ^1^H-NMR and ^13^C-NMR, see Table 1; positive ESIMS *m/z* 418 [M+Na]^+^; positive HRESIMS [M+Na]^+^ *m/z* 418.1632 (calcd for C_23_H_25_NO_5_Na, 418.1630).

*Daphnicyclidin N* (**2**): amorphous light yellow powder; 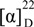 −65.5 (*c* 0.11, MeOH); UV (MeOH) λ_max_ (log ε) 281 (1.14), 204 (5.00) nm; IR (KBr) *ν*_max_ 3425, 2920, 1686, 1643, 1616, 1525, 1456, 1385, 1126, 991 cm^−1^; ^1^H-NMR and ^13^C-NMR, see Table 1; positive ESIMS *m/z* 448 [M+Na]^+^; positive HRESIMS [M+Na]^+^ *m/z* 448.1738 (calcd for C_24_H_27_NO_6_Na, 448.1736).

*Calyciphylline Q* (**3**): light yellow oil; 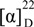 −16.8 (*c* 0.08, MeOH); UV (MeOH) λ_max_ (log ε) 280 (4.96) nm; IR (KBr) *ν*_max_ 2925, 1709, 1685, 1615, 1440, 1378, 1096 cm^−1^; ^1^H-NMR and ^13^C-NMR, see Table 1; positive ESIMS *m/z* 388 [M+Na]^+^; positive HRESIMS [M+Na]^+^ *m/z* 388.1890 (calcd for C_23_H_27_NO_3_Na, 388.1889).

*Calyciphylline R* (**4**): light yellow oil; 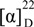 −44.7 (*c* 0.15, MeOH); UV (MeOH) λ_max_ (log ε) 270.5 (4.23), 204.5 (4.88) nm; IR (KBr) *ν*_max_ 2920, 1745, 1695, 1575, 1445, 1380 cm^−1^; ^1^H-NMR and ^13^C-NMR, see Table 1; positive ESIMS *m/z* 342 [M+H]^+^; positive HRESIMS [M+H]^+^ *m/z* 342.2068 (calcd for C_21_H_28_NO_3_, 342.2069).

*Calyciphylline S* (**5**): light yellow oil; 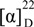 −81.2 (*c* 0.13, DMSO); UV (MeOH) λ_max_ (log ε) 247.5 (4.54) nm; IR (KBr) *ν*_max_ 3425, 2920, 1701, 1675, 1611, 1438, 1380, 1226, 995, 565 cm^−1^; ^1^H-NMR and ^13^C-NMR, see Table 1; positive ESIMS *m/z* 360 [M+H]^+^; positive HRESIMS *m/z* [M+H]^+^ 360.2172 (calcd for C_21_H_30_NO_4_, 360.2175).

### 3.4. Assays for In Vitro Antitumor Activity

The cytotoxicity of compounds **1**–**9** against mouse lymphocytic leukemia P-388 cells, human lung carcinoma A-549 cells, human promyelocytic leukemia HL-60 cells and human gastric carcinoma SGC-7901 cells was evaluated by 3-(4,5-dimethylthiazol-2-yl)-2,5-diphenyltetrazolium bromide (MTT) colorimetric assay method *in vitro*. All cells were cultured in RPMI-1640 medium supplemented with 10% fetal bovine serum, 100 U/mL benzyl penicillin, and 100 U/mL streptomycin at 37 C in a humidified atmosphere with 5% CO_2_. The logarithmic phase cells were seeded on 96-well plates at the concentration of 4 × 10^3^ cell/mL and incubated with various concentrations (100, 80, 60, 40, 20, 10, 1 and 0.25 μM in medium containing less than 0.1% DMSO) of test compounds in triples wells for 48 h, and cisplatin was used as positive control. After that, 20 μL MTT (5 mg/mL) was added to each well, and incubated for another 4 h. The water-insoluble dark blue formazan crystals formed during MTT cleavage in actively metabolizing cells were dissolved in DMSO. The optical density of each well was measured with a Bio-Rad 680 microplate reader at 570 nm. Cytotoxicity was expressed as the concentration of drug inhibiting cell growth by 50% (IC_50_).

## 4. Conclusions

Phytochemical investigation of the stem bark of *Daphniphyllum macropodum*, lead to the isolation of five new *Daphniphyllum* alkaloids **1**–**5**, along with four known ones **6**–**9**. Their structures and relative configurations were elucidated on the basis of spectroscopic methods, especially 2D NMR techniques. All of the compounds were tested for cytotoxic activity against P-388, A-549, HL-60 and SGC-7901 cell lines. P-388 cells were sensitive to compounds **1**, **2**, **8** and **9**, which exhibited selective cytotoxic activity with IC_50_ values of 5.7, 6.5, 10.3 and 13.8 µM, respectively. Interestingly, compounds **1** and **2** also showed a moderate cytotoxic activity against SGC-7901 cells with IC_50_ values of 22.4 and 25.6 µM. These preliminary results suggested that the cytotoxicity of these compounds appeared to be structure dependent, indicating that *Daphniphyllum* alkaloids of the **1** and **2** structural type possessed the potential for further investigation.

## Supplementary Materials

Supplementary materials can be accessed at: http://www.mdpi.com/1420-3049/19/3/3055/s1.
