# Supplementary material for: Five New Alkaloids from the Stem Bark of Daphniphyllum macropodum
Source: Molecules. 2014 Mar 10;19(3):3055–67. doi: 10.3390/molecules19033055 (PMC6271962; doi:10.3390/molecules19033055)

# Supporting Information

**Figure S1.** Selected 2D NMR correlations of daphnicyclidin N (**2**).

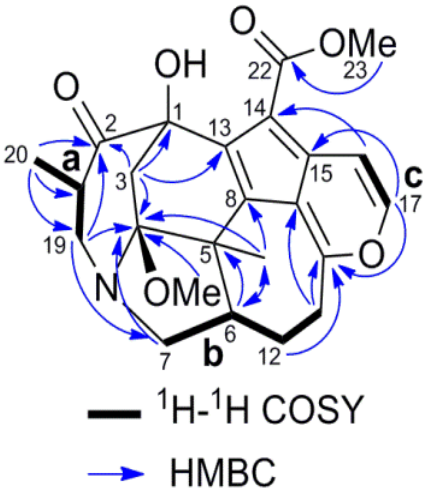


**Figure S2.** Selected 2D NMR correlations of calyciphylline R (**4**).

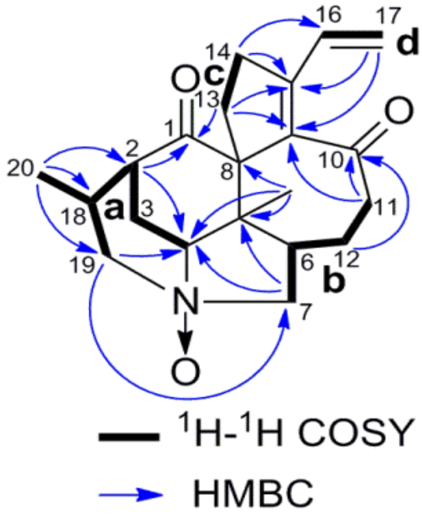


**Figure S3.** Selected 2D NMR correlations of calyciphylline S (**5**).

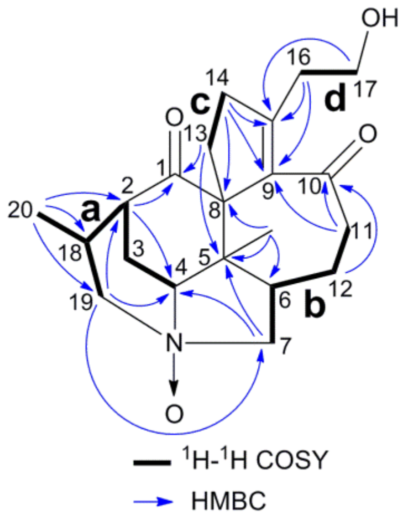

**Figure S4.** $^1$H-NMR (500 MHz, $CD_3OD$) spectrum of daphnicyclidin M (**1**).

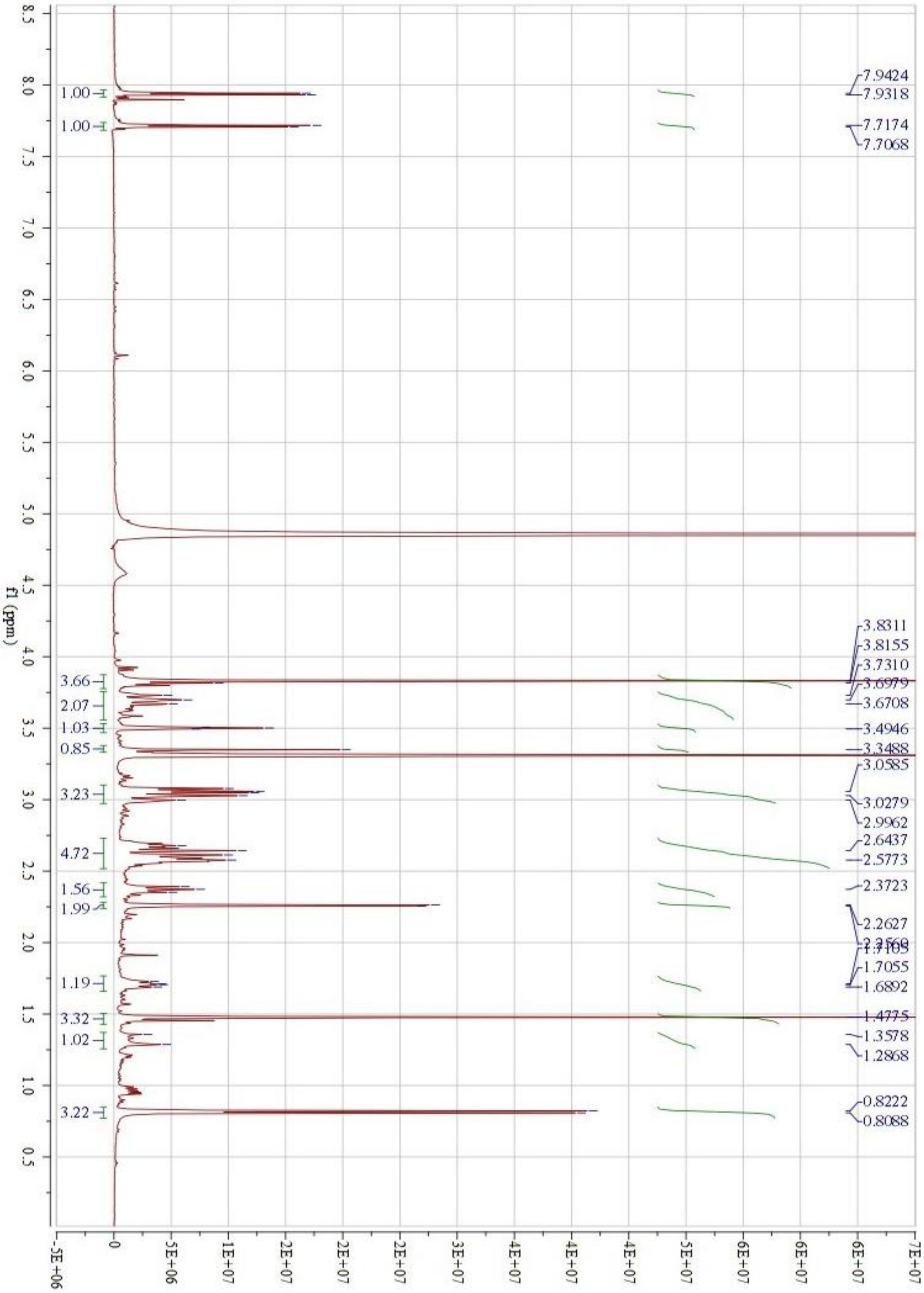

**Figure S5.** $^{13}$C-NMR (125 MHz, $CD_3OD$) spectrum of daphnicyclidin M (**1**).

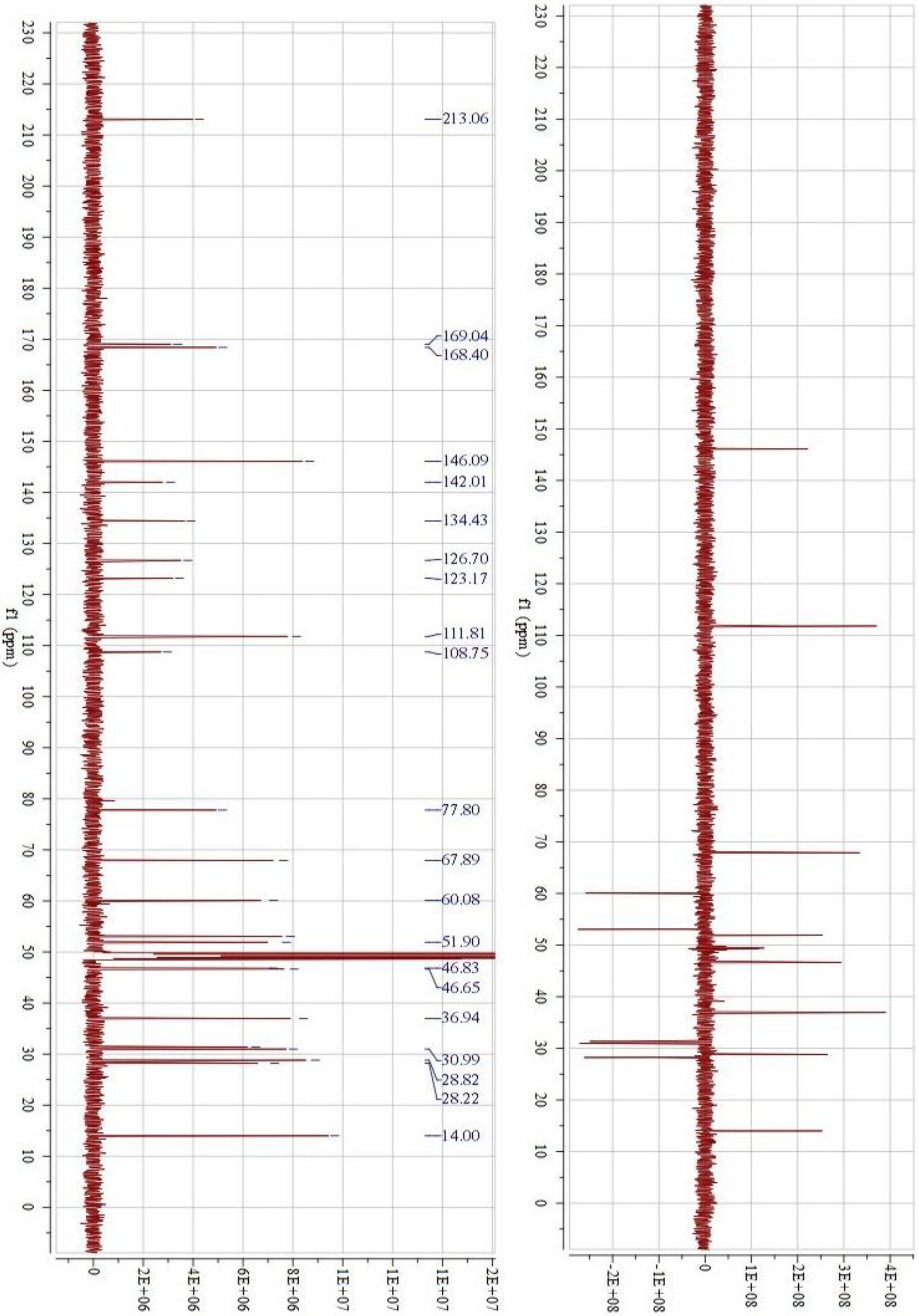

**Figure S6.** HSQC spectrum of daphnicyclidin M (**1**) in $CD_3OD$.

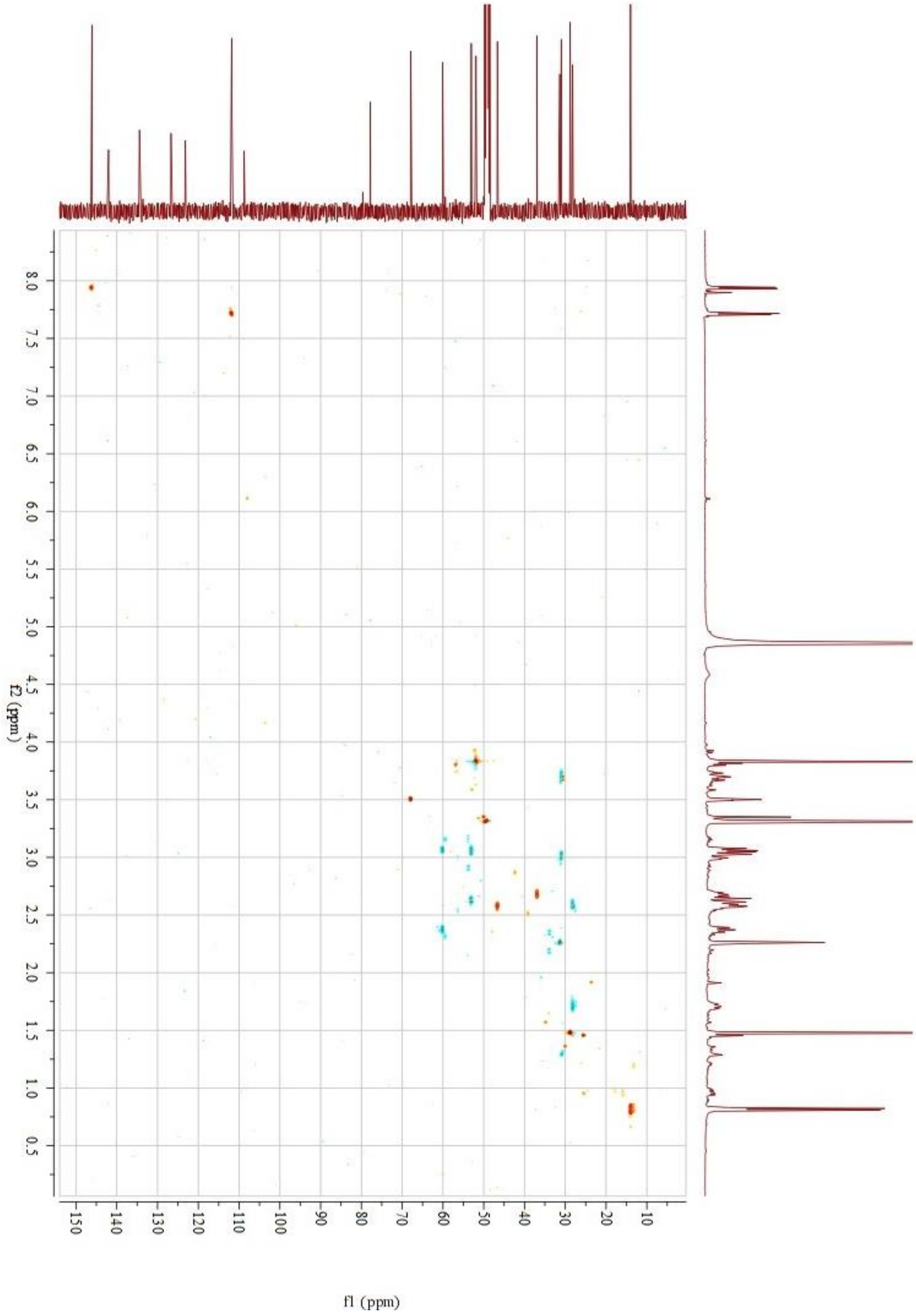

**Figure S7.** $^{1}$H-$^{1}$H COSY spectrum of daphnicyclidin M (**1**) in $CD_3OD$.

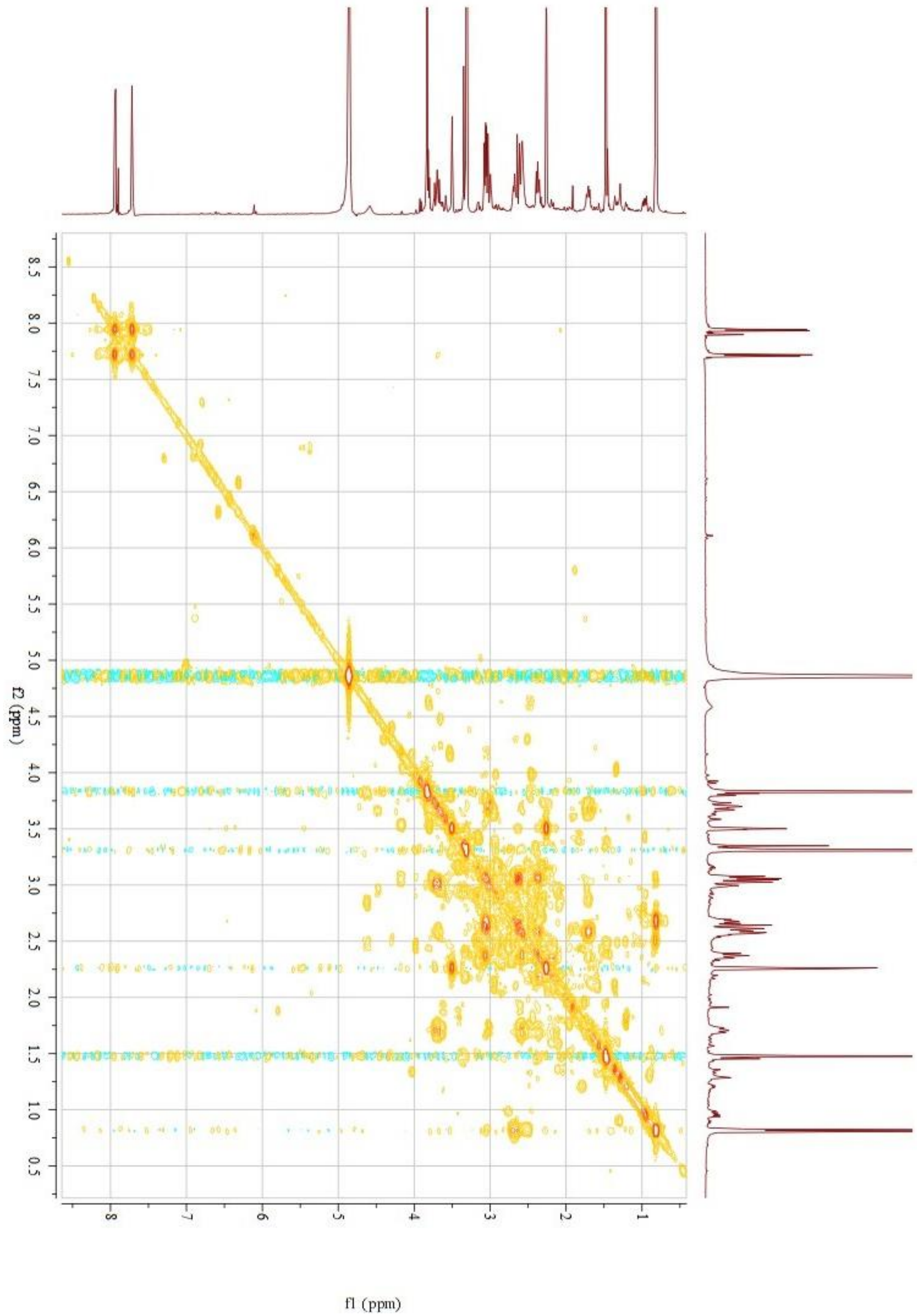

**Figure S8.** HMBC spectrum of daphnicyclidin M (**1**) in $CD_3OD$.

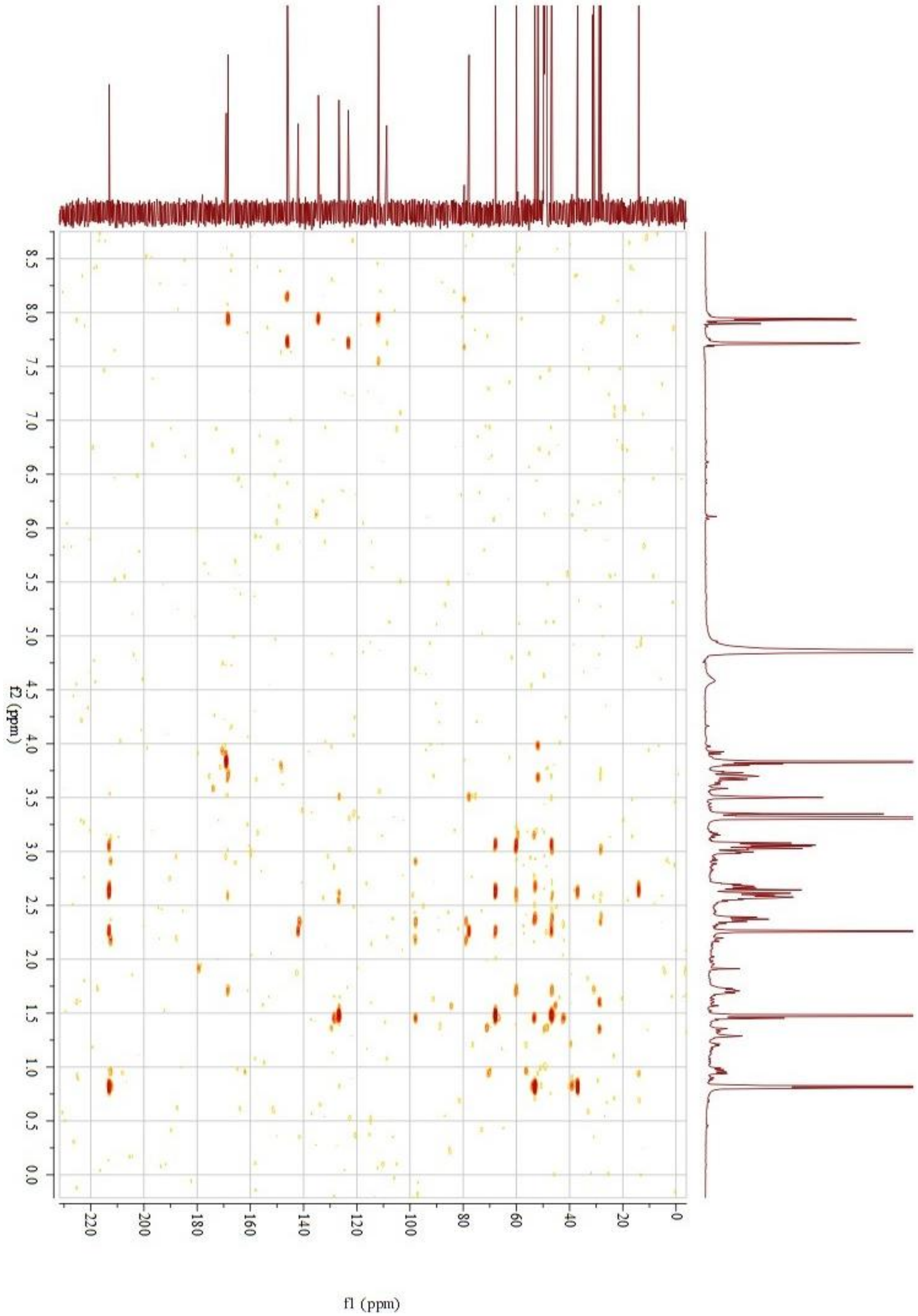

**Figure S9.** NOESY spectrum of daphnicyclidin M (**1**) in $CD_3OD$.

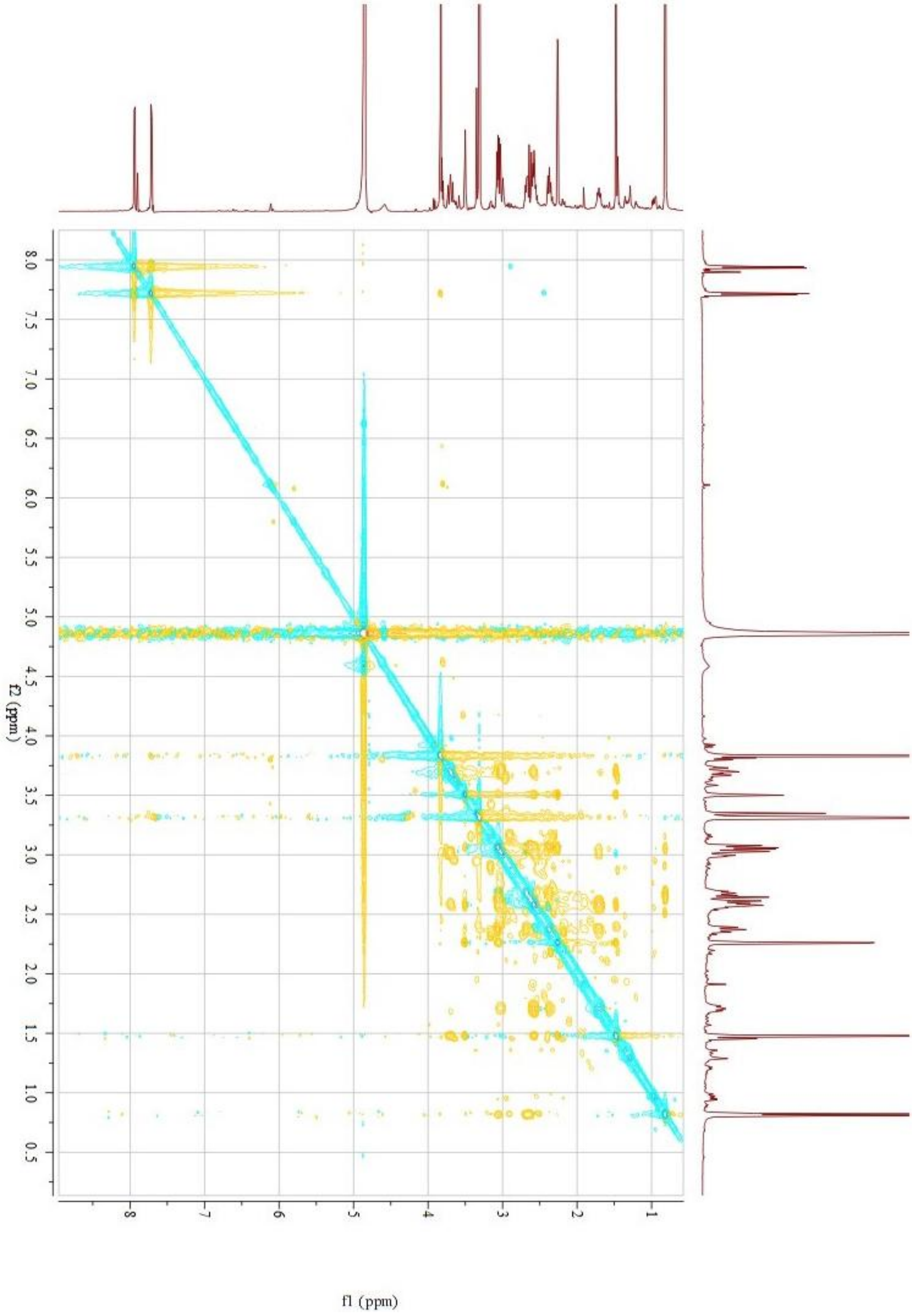

**Figure S10.** ESIMS spectrum of daphnicyclidin M (**1**).

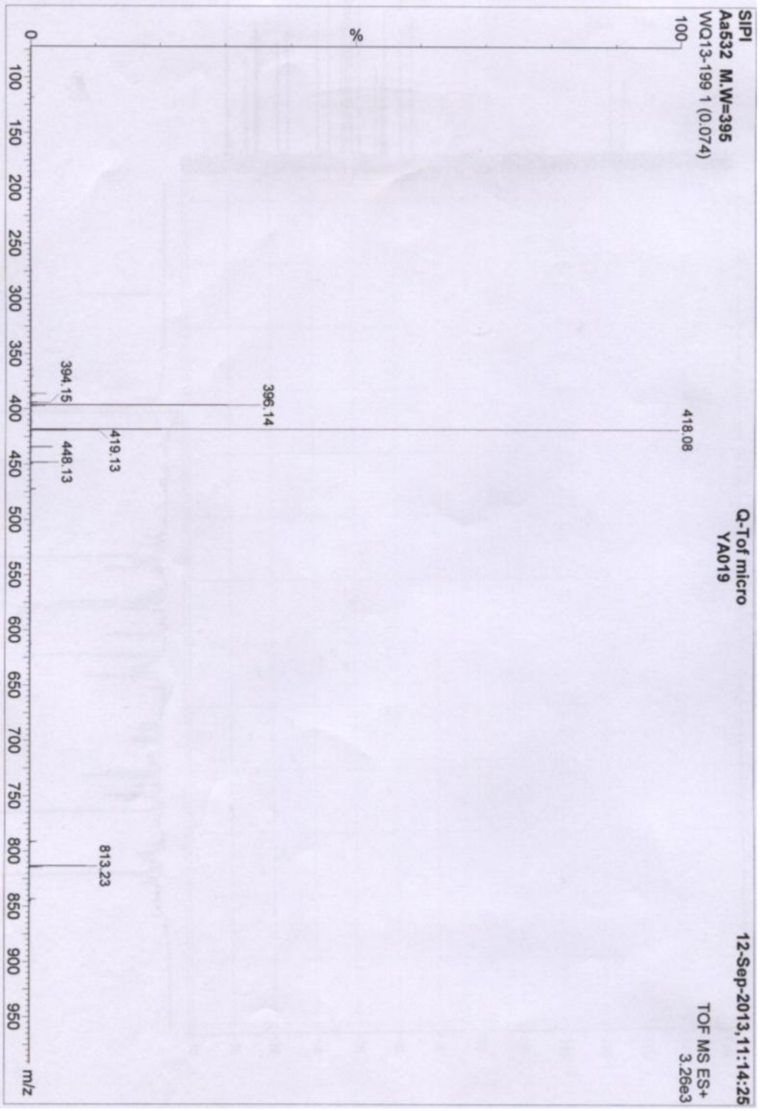


**Figure S11.** HRESIMS spectrum of daphnicyclidin M (**1**).

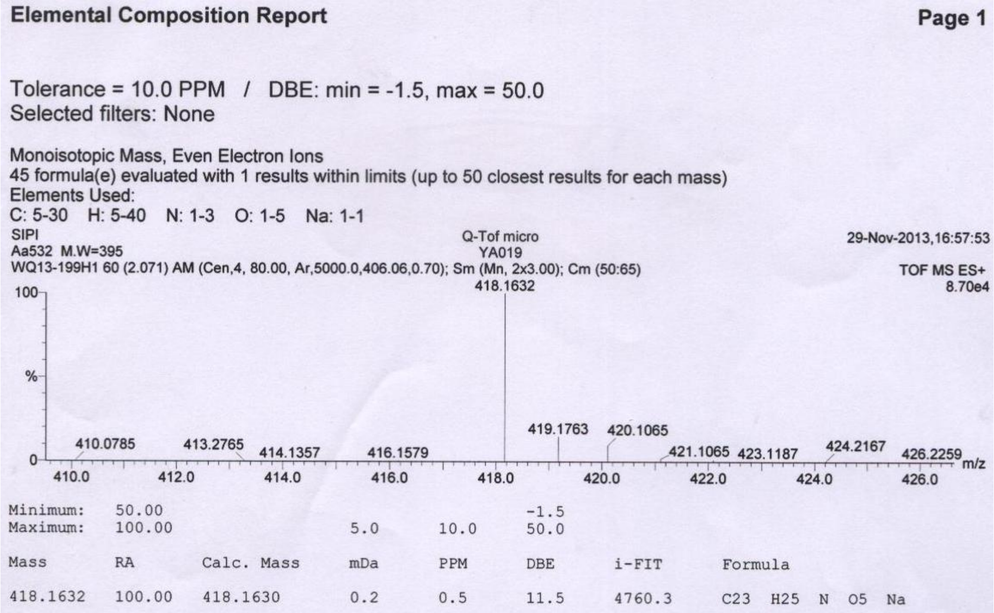
**Elemental Composition Report** **Page 1**

Tolerance = 10.0 PPM / DBE: min = -1.5, max = 50.0
Selected filters: None

Monoisotopic Mass, Even Electron Ions
45 formula(e) evaluated with 1 results within limits (up to 50 closest results for each mass)
Elements Used:
C: 5-30 H: 5-40 N: 1-3 O: 1-5 Na: 1-1

SIPI
Aa532 M.W=395
WQ13-199H1 60 (2.071) AM (Cen,4, 80.00, Ar,5000.0,406.06,0.70); Sm (Mn, 2x3.00); Cm (50:65)
Q-Tof micro
YA019
29-Nov-2013,16:57:53
TOF MS ES+
8.70e4



| | | | | | | | |
|---|---|---|---|---|---|---|---|
| Minimum: | 50.00 | | | | -1.5 | | |
| Maximum: | 100.00 | | 5.0 | 10.0 | 50.0 | | |
| Mass | RA | Calc. Mass | mDa | PPM | DBE | i-FIT | Formula |
| 418.1632 | 100.00 | 418.1630 | 0.2 | 0.5 | 11.5 | 4760.3 | C23 H25 N O5 Na |

**Figure S12.** $^1H$-NMR (500 MHz, $CD_3OD$) spectrum of daphnicyclidin N (**2**).

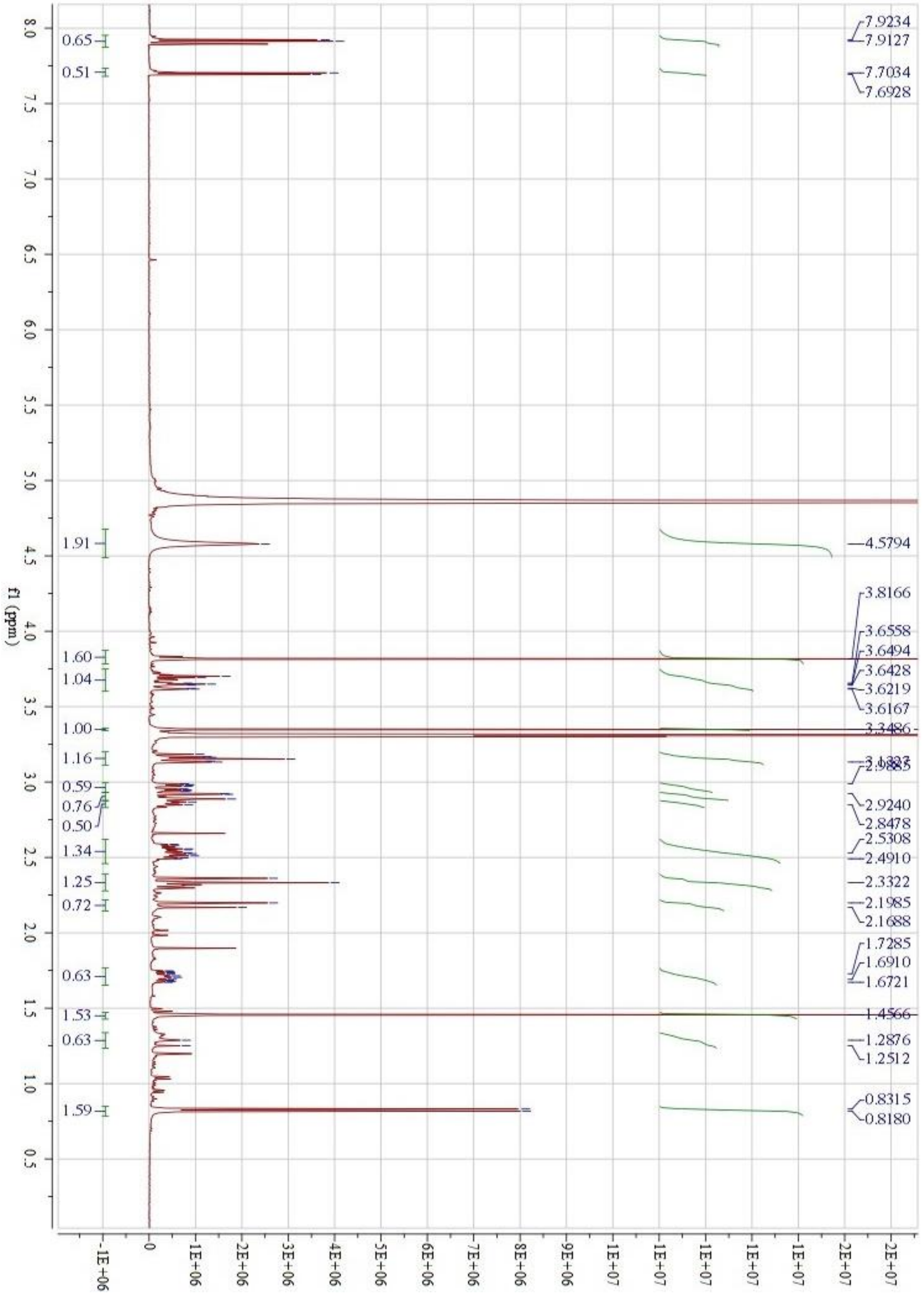

**Figure S13.** $^{13}$C-NMR (125 MHz, $CD_3OD$) spectrum of daphnicyclidin N (**2**).

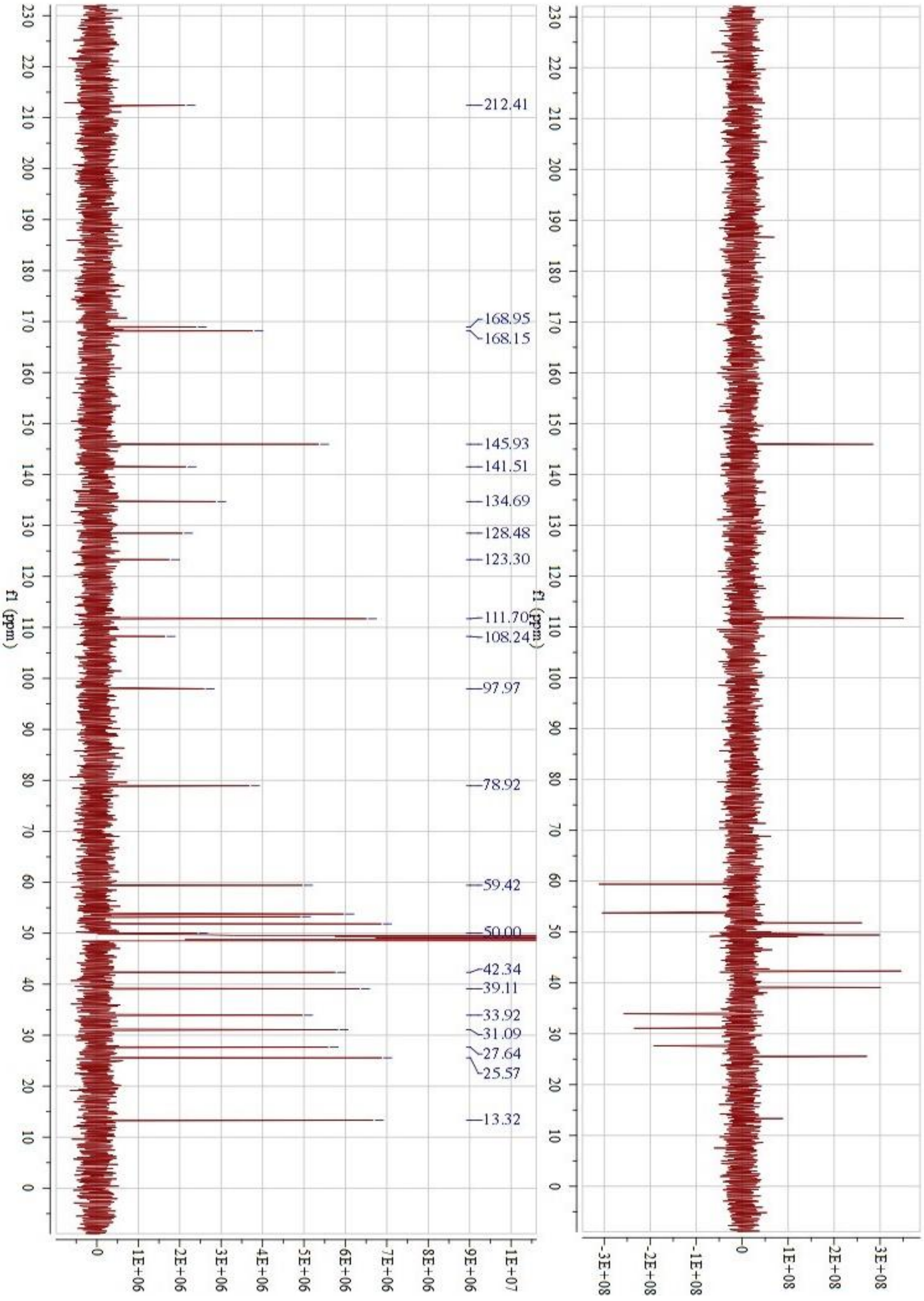

**Figure S14.** HSQC spectrum of daphnicyclidin N (**2**) in $CD_3OD$.

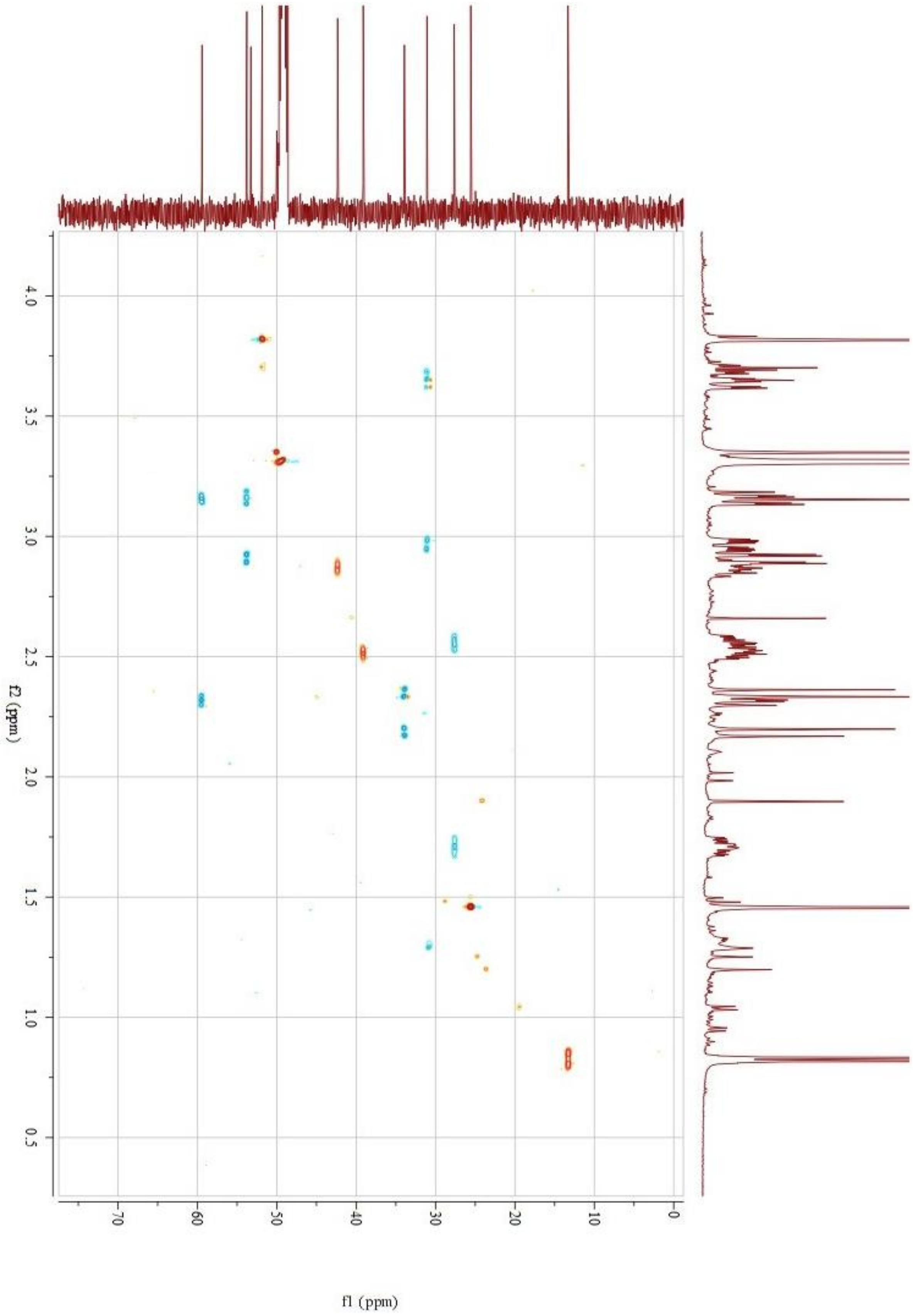

**Figure S15.** $^1H$-$^1H$ COSY spectrum of daphnicyclidin N (**2**) in $CD_3OD$.

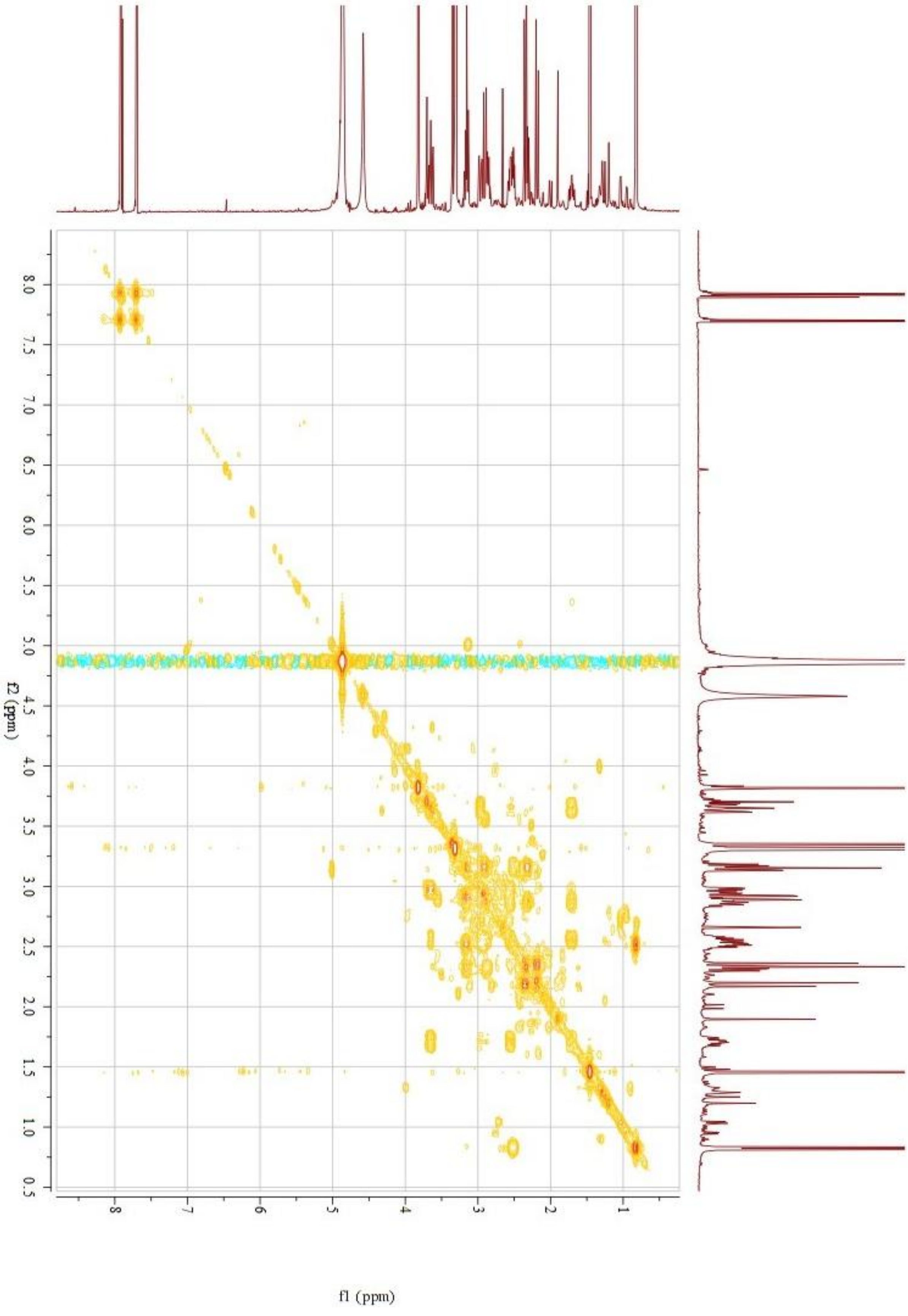

**Figure S16.** HMBC spectrum of daphnicyclidin N (**2**) in $CD_3OD$.

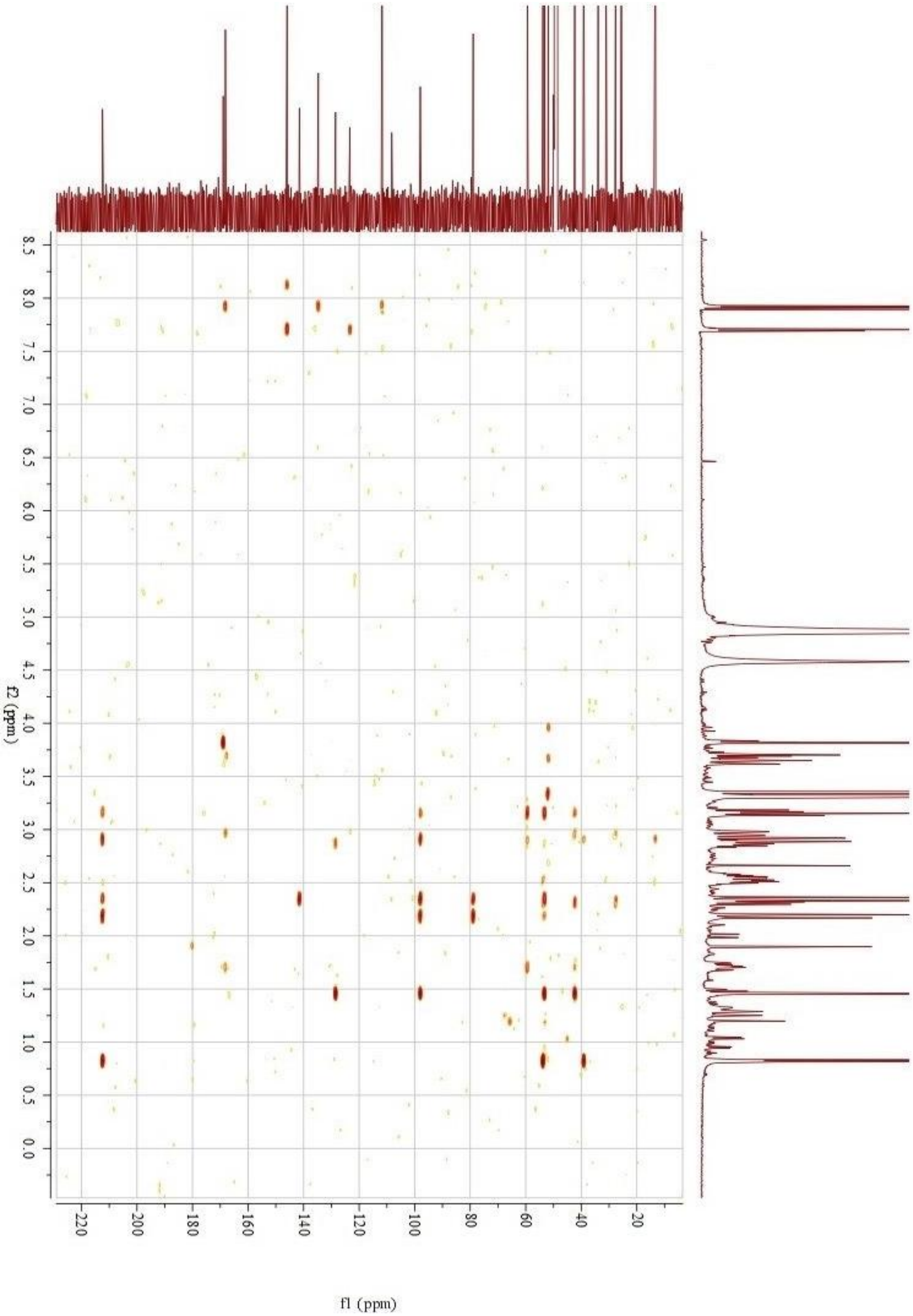

**Figure S17.** NOESY spectrum of daphnicyclidin N (**2**) in $CD_3OD$.

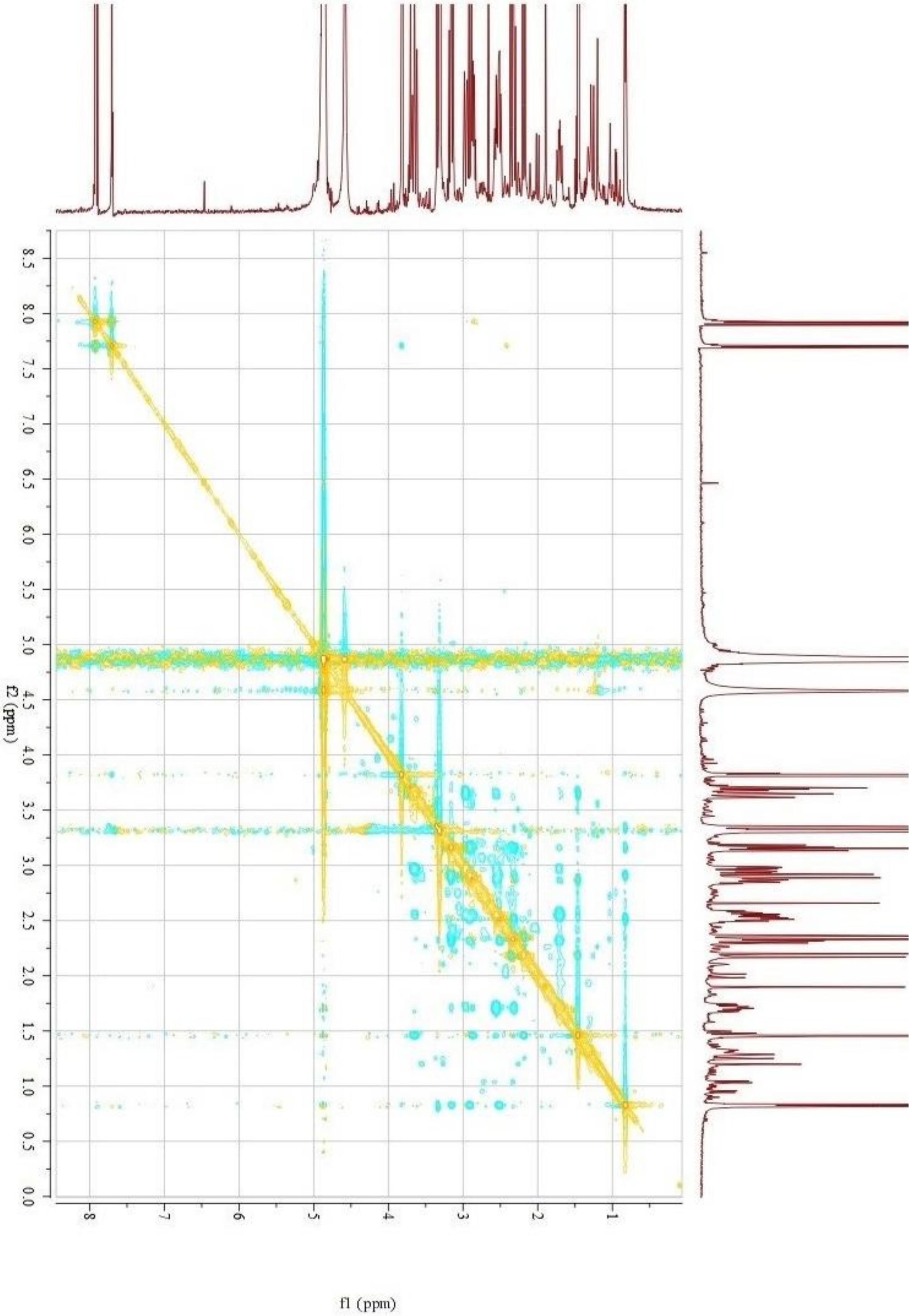

**Figure S18.** ESIMS spectrum of daphnicyclidin N (**2**).

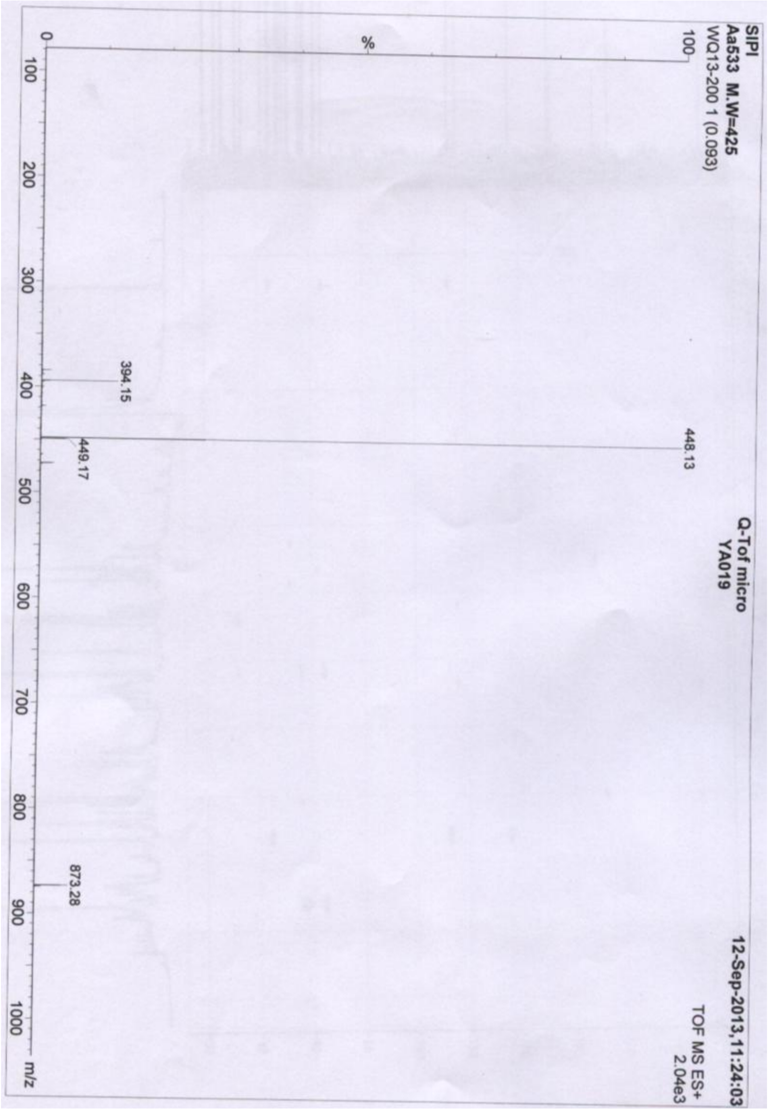


**Figure S19.** HRESIMS spectrum of daphnicyclidin N (2).

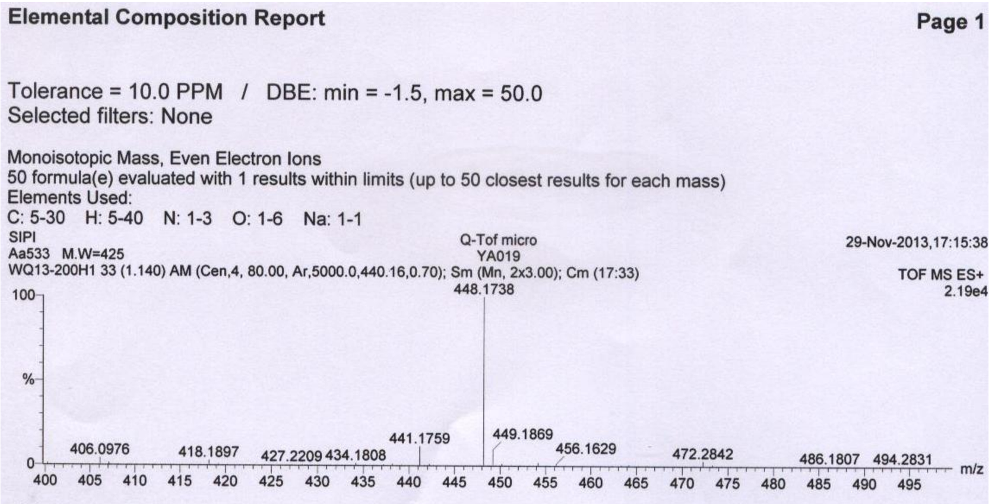


| Mass | RA | Calc. Mass | mDa | PPM | DBE | i-FIT | Formula |
|---|---|---|---|---|---|---|---|
| Minimum: | 50.00 | | | | -1.5 | | |
| Maximum: | 100.00 | | 5.0 | 10.0 | 50.0 | | |
| 448.1738 | 100.00 | 448.1736 | 0.2 | 0.4 | 11.5 | 2896.1 | C24 H27 N O6 Na |

**Figure S20.** $^{1}$H-NMR (500 MHz, $CD_3OD$) spectrum of calyciphylline Q (**3**).

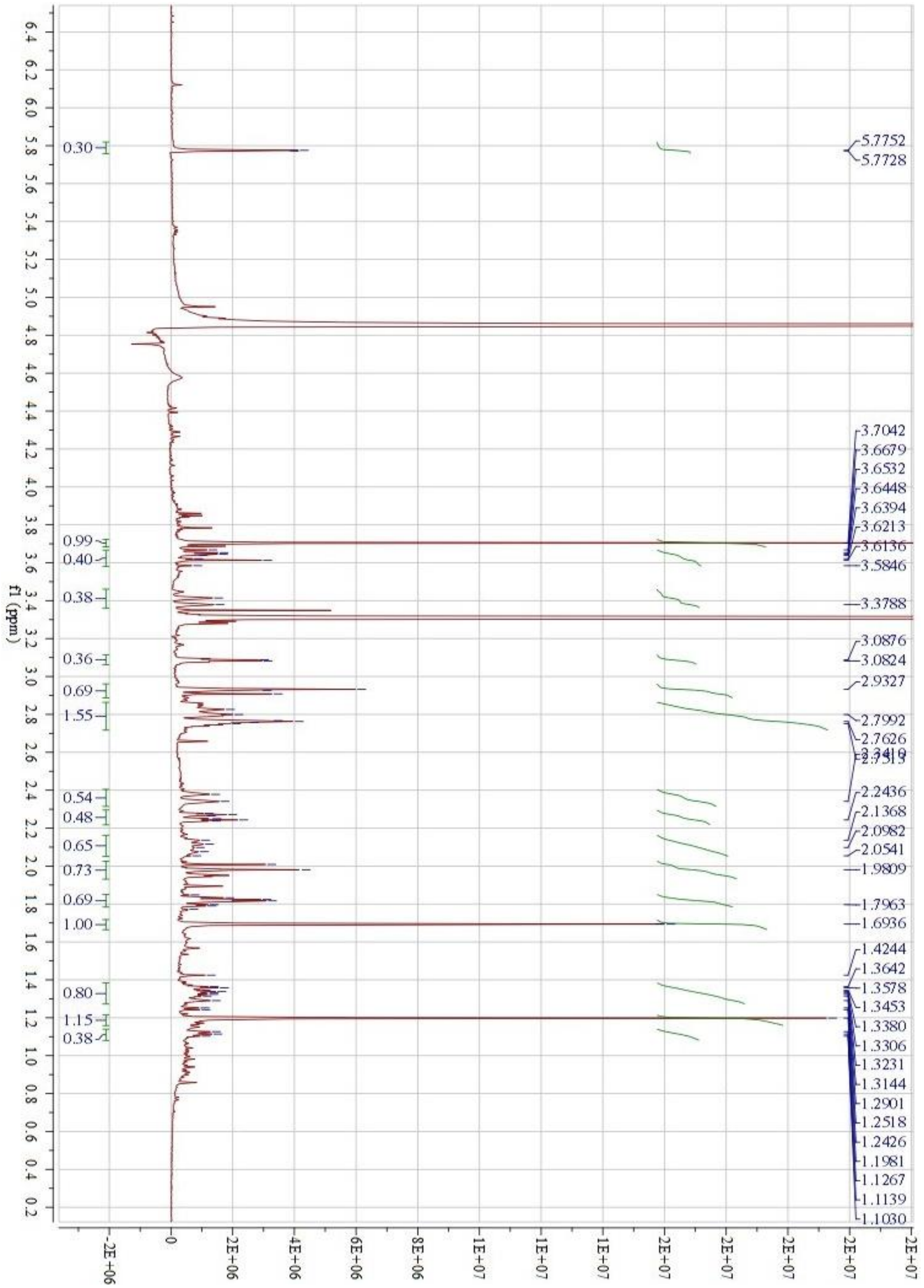

**Figure S21.** $^{13}$C-NMR (125 MHz, $CD_3OD$) spectrum of calyciphylline Q (**3**).

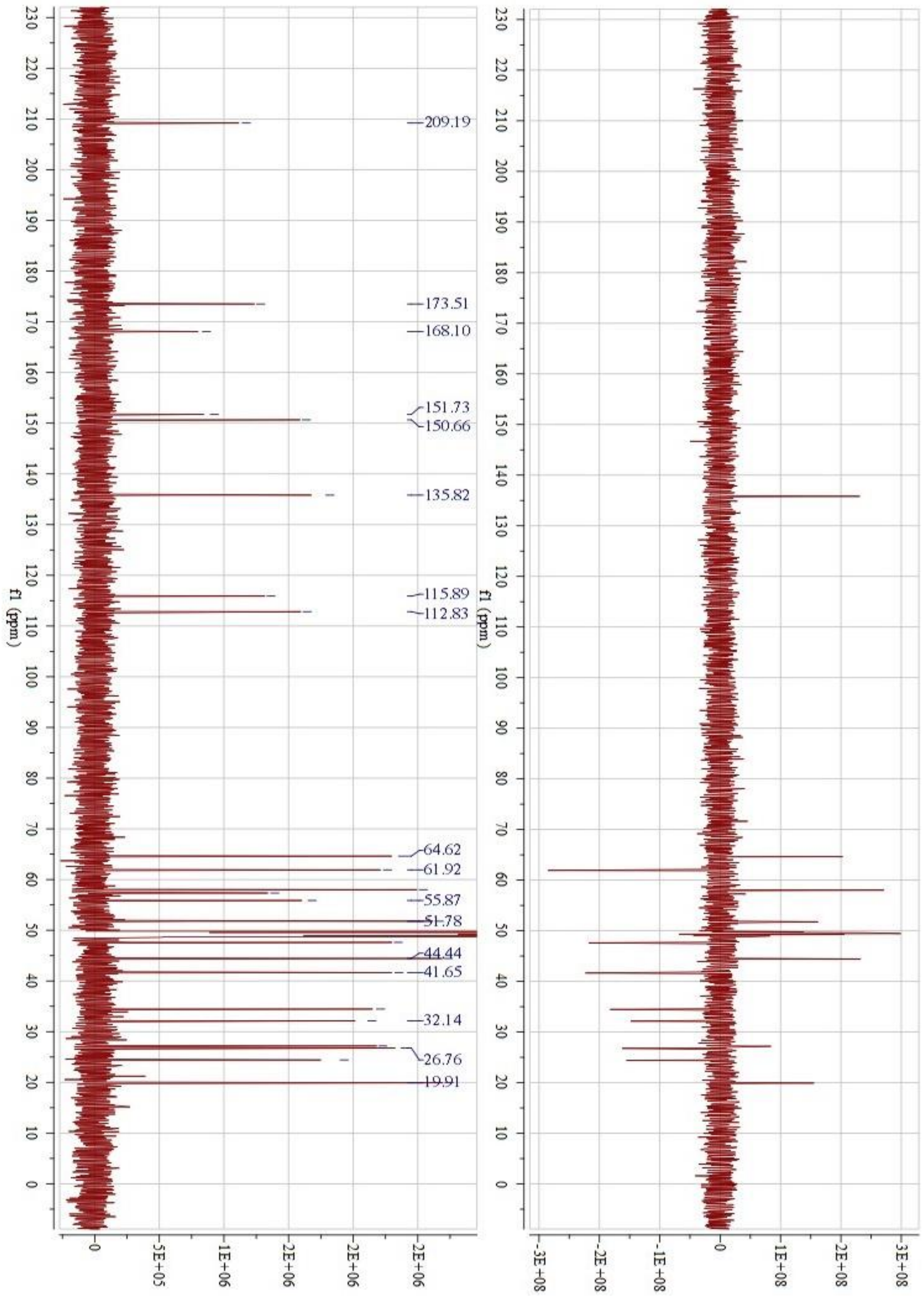

**Figure S22.** HSQC spectrum of calyciphylline Q (**3**) in $CD_3OD$.

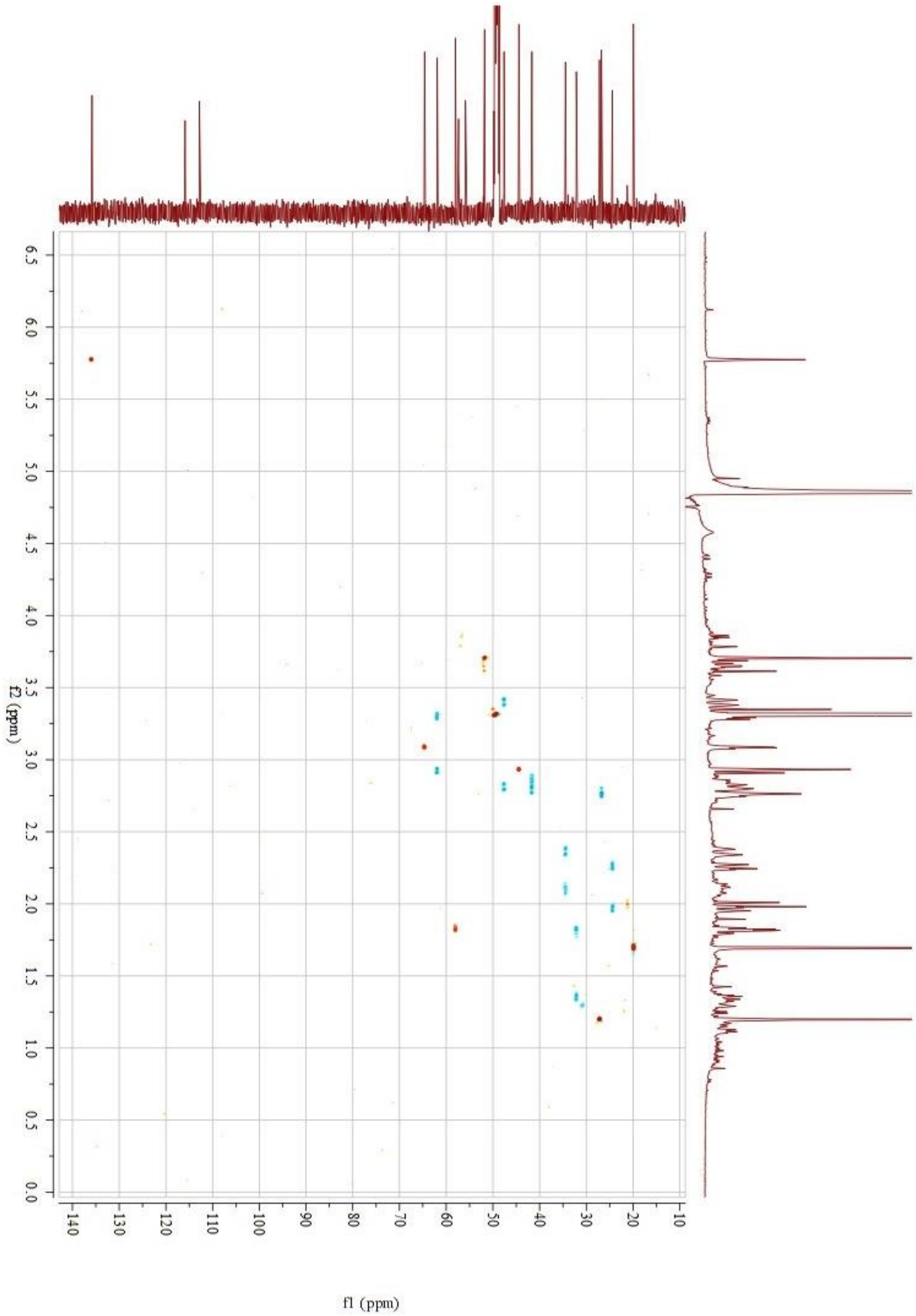

**Figure S23.** $^{1}$H-$^{1}$H COSY spectrum of calyciphylline Q (**3**) in $CD_3OD$.

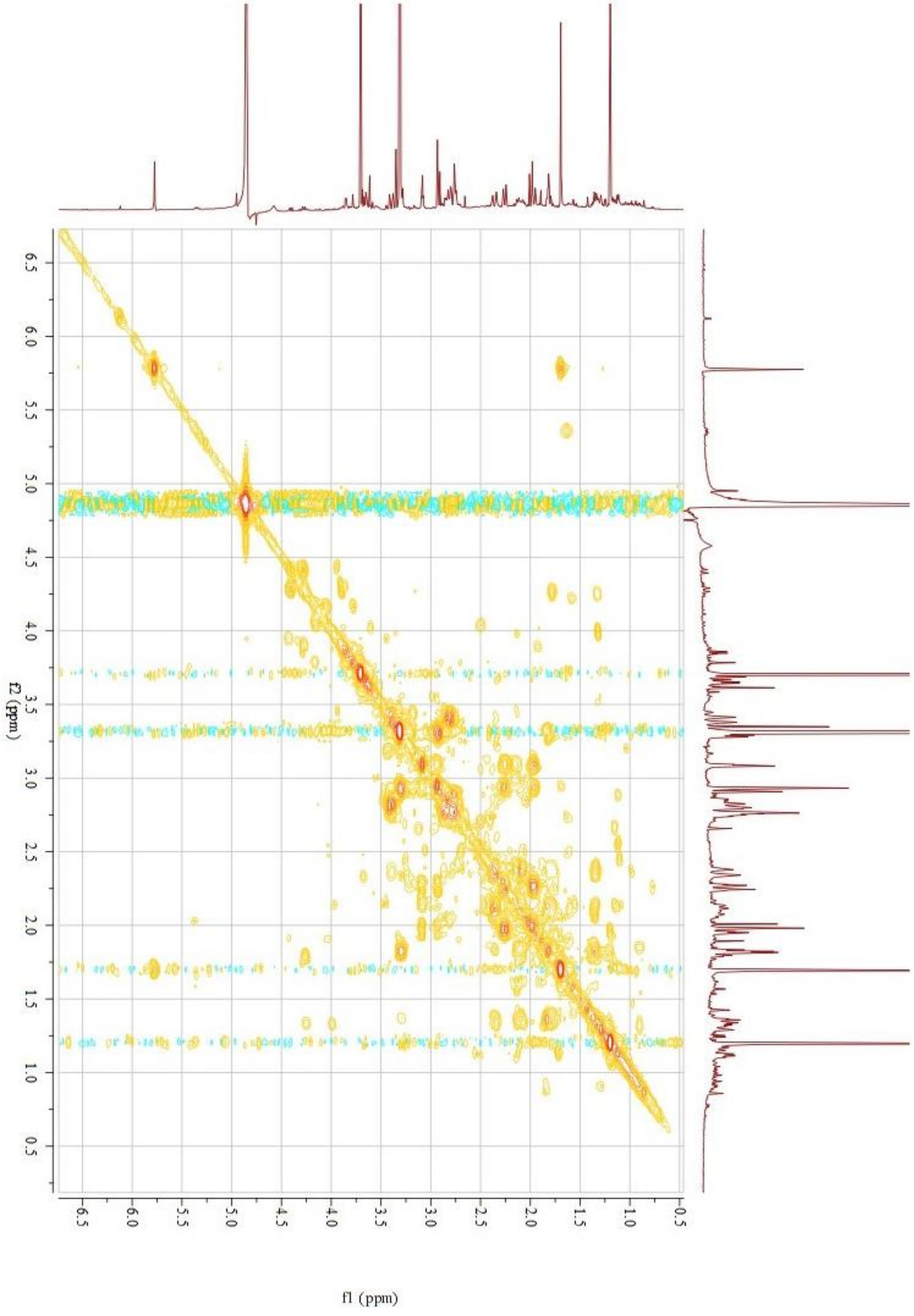

**Figure S24.** HMBC spectrum of calyciphylline Q (**3**) in $CD_3OD$.

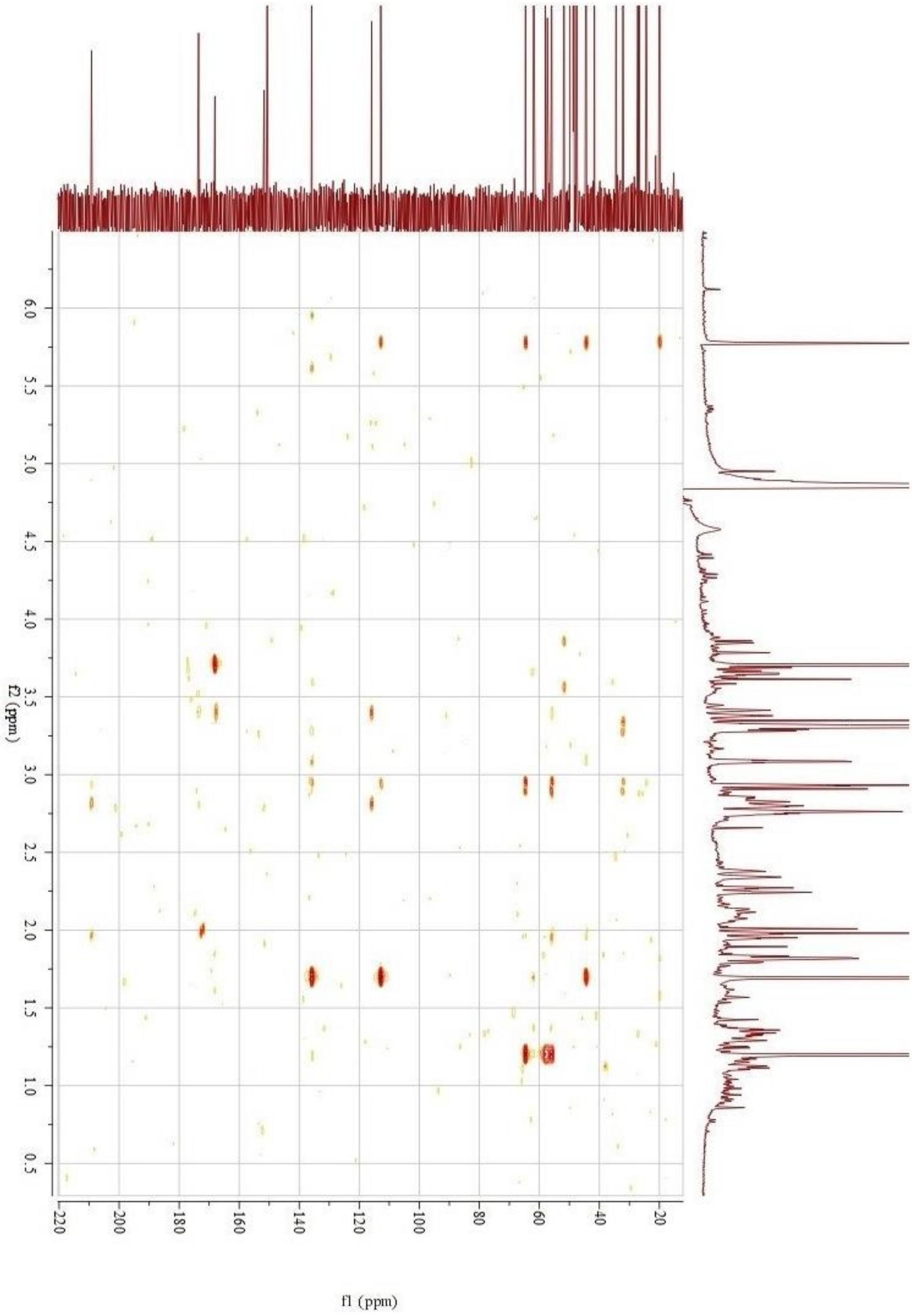

**Figure S25.** NOESY spectrum of calyciphylline Q (**3**) in $CD_3OD$.

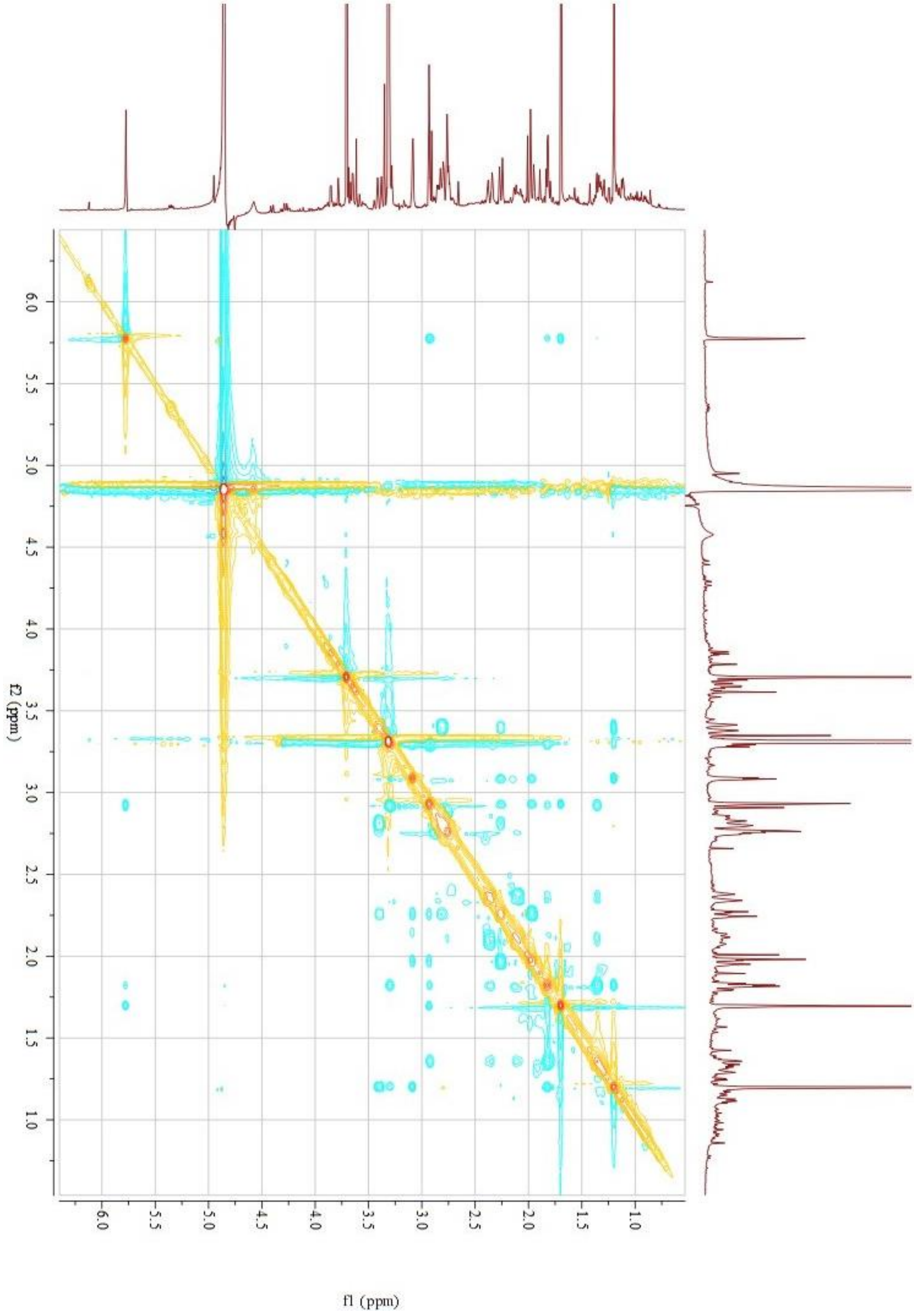

**Figure S26.** ESIMS spectrum of calyciphylline Q (**3**).

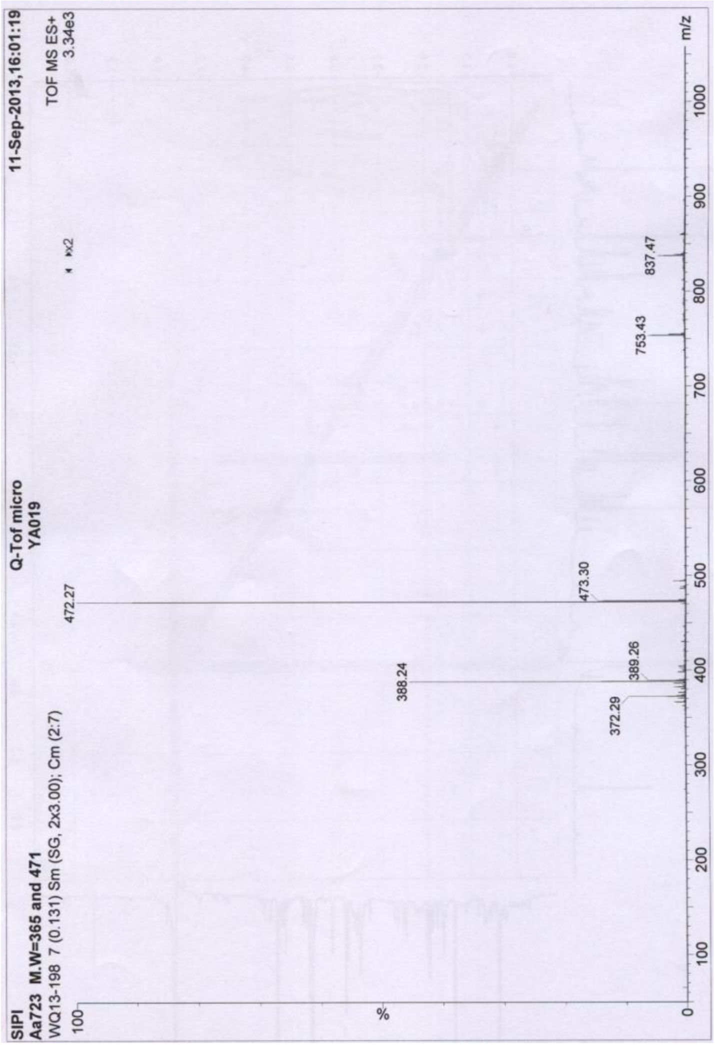


**Figure S27.** HRESIMS spectrum of calyciphylline Q (**3**).

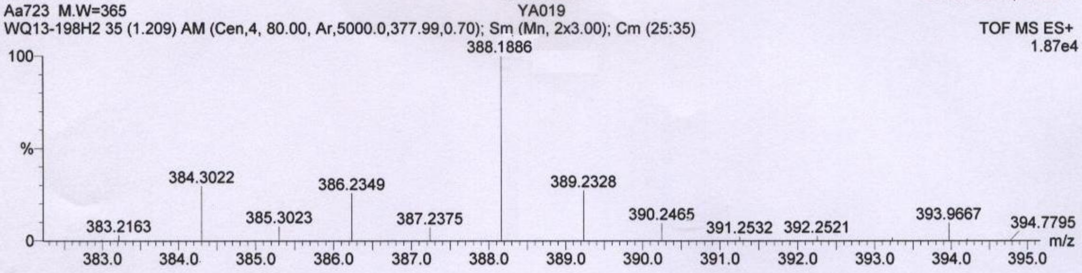

**Elemental Composition Report** **Page 1**

Tolerance = 10.0 PPM / DBE: min = -1.5, max = 50.0
Selected filters: None

Monoisotopic Mass, Even Electron Ions
45 formula(e) evaluated with 2 results within limits (up to 50 closest results for each mass)
Elements Used:
C: 5-30 H: 5-40 N: 1-3 O: 1-5 Na: 1-1



| | | | | | | | |
|---|---|---|---|---|---|---|---|
| Minimum: | 50.00 | | | | -1.5 | | |
| Maximum: | 100.00 | | 5.0 | 10.0 | 50.0 | | |
| Mass | RA | Calc. Mass | mDa | PPM | DBE | i-FIT | Formula |
| 388.1886 | 100.00 | 388.1889 | -0.1 | -0.3 | 9.5 | 281.4 | C23 H27 N O3 Na |

**Figure S28.** $^{1}$H-NMR (500 MHz, $CD_3OD$) spectrum of calyciphylline R (**4**).

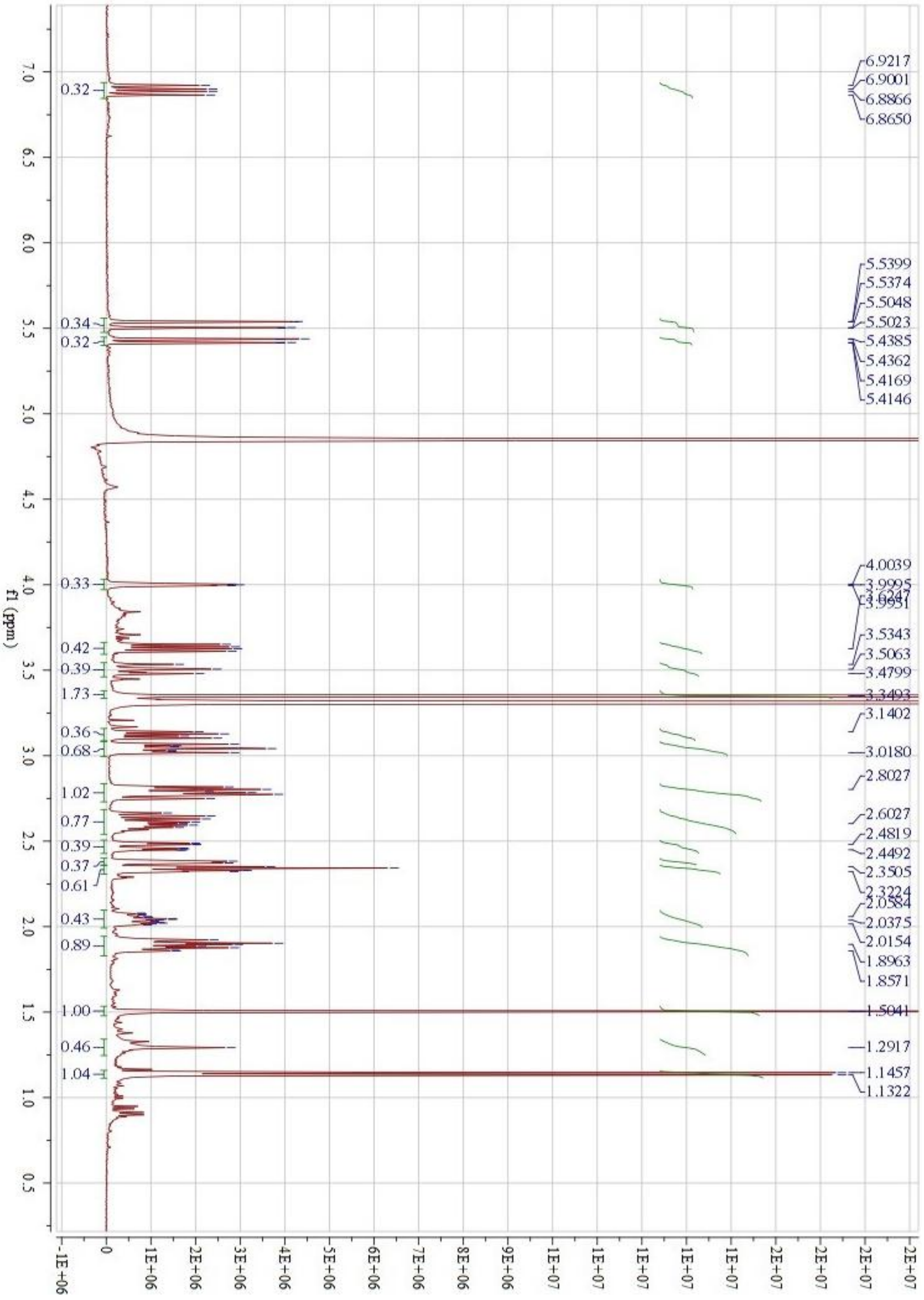

**Figure S29.** $^{13}$C-NMR (125 MHz, $CD_3OD$) spectrum of calyciphylline R (**4**).

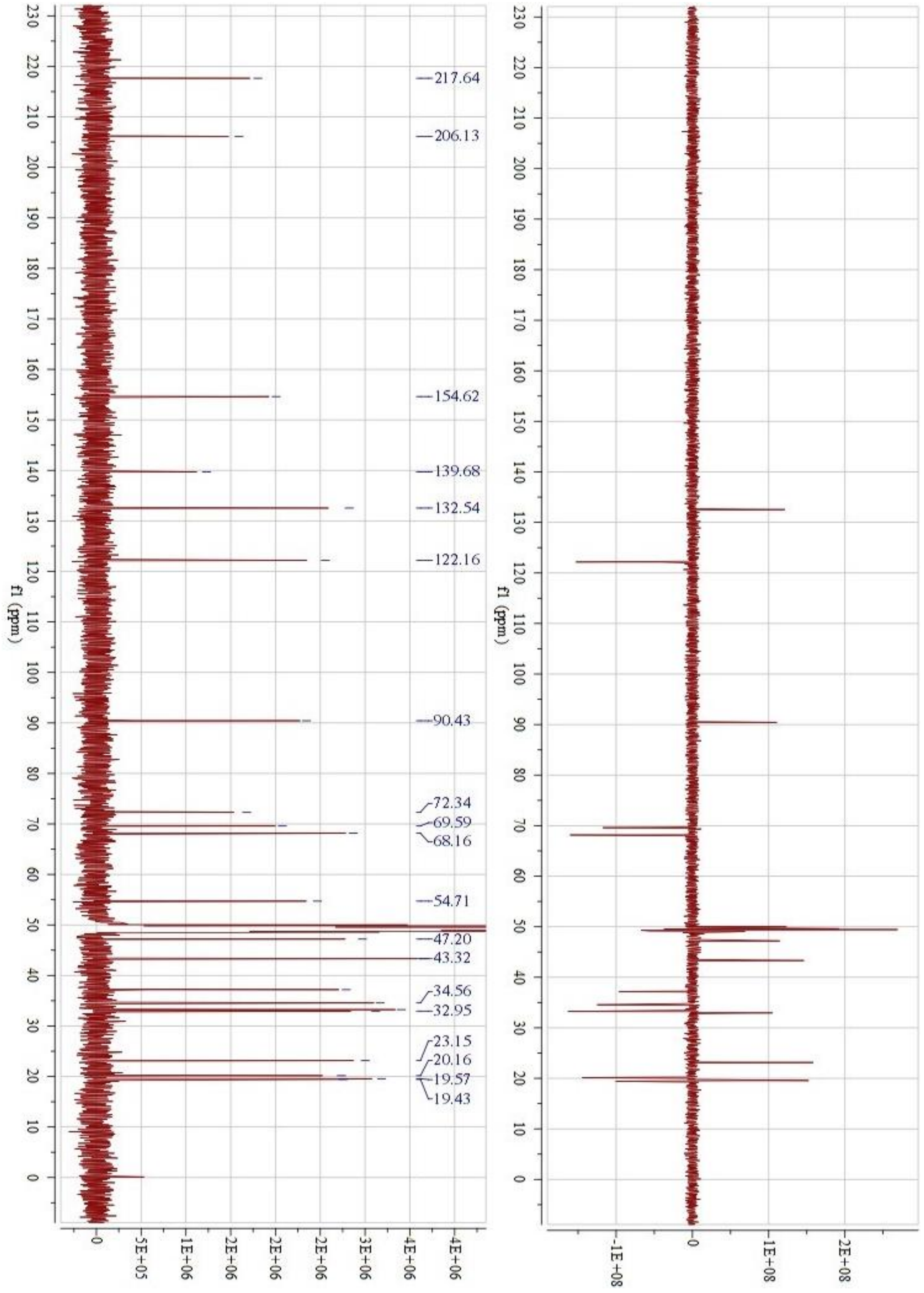

**Figure S30.** HSQC spectrum of calyciphylline R (**4**) in $CD_3OD$.

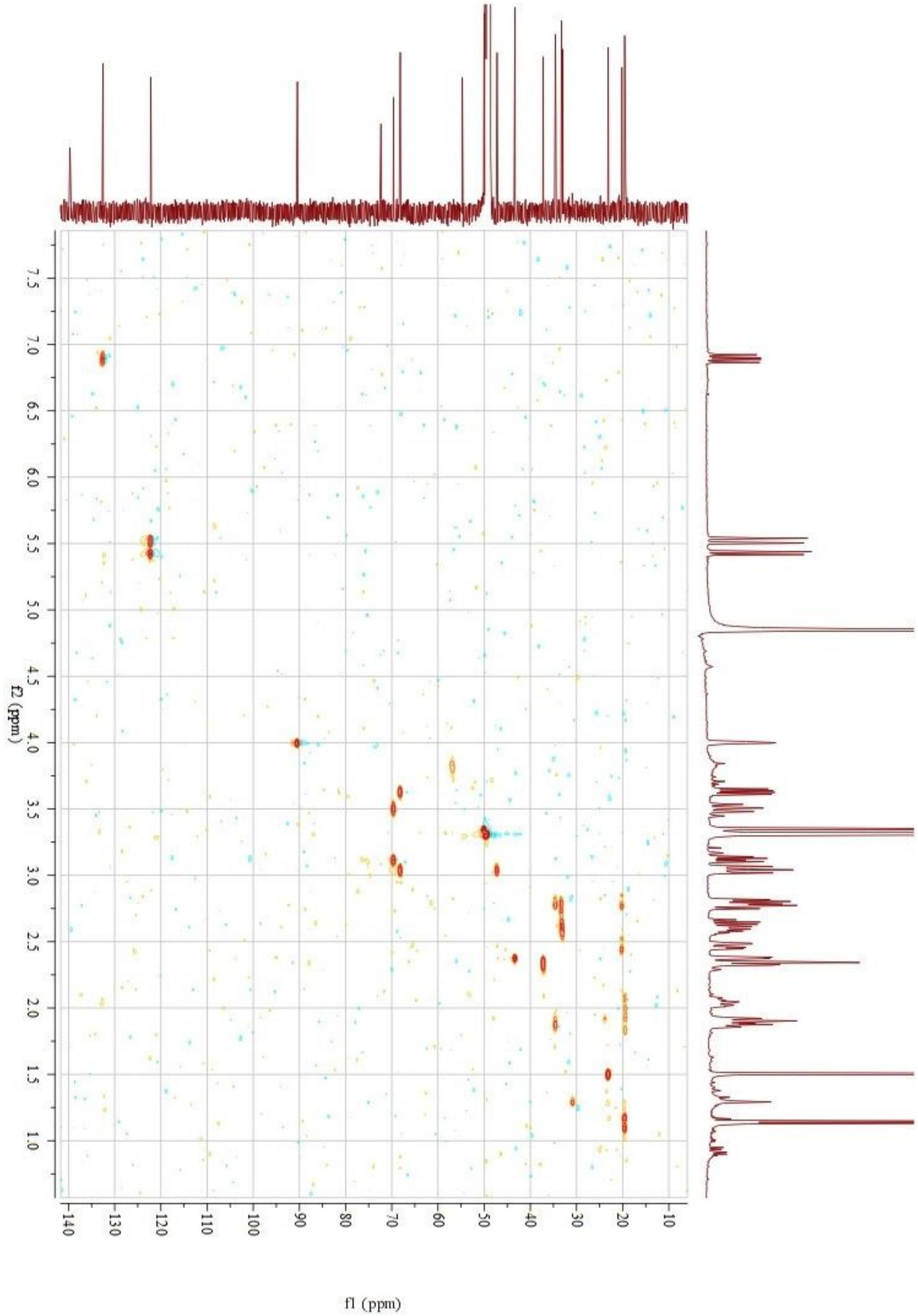

**Figure S31.** $^{1}$H-$^{1}$H COSY spectrum of calyciphylline R (**4**) in $CD_3OD$.

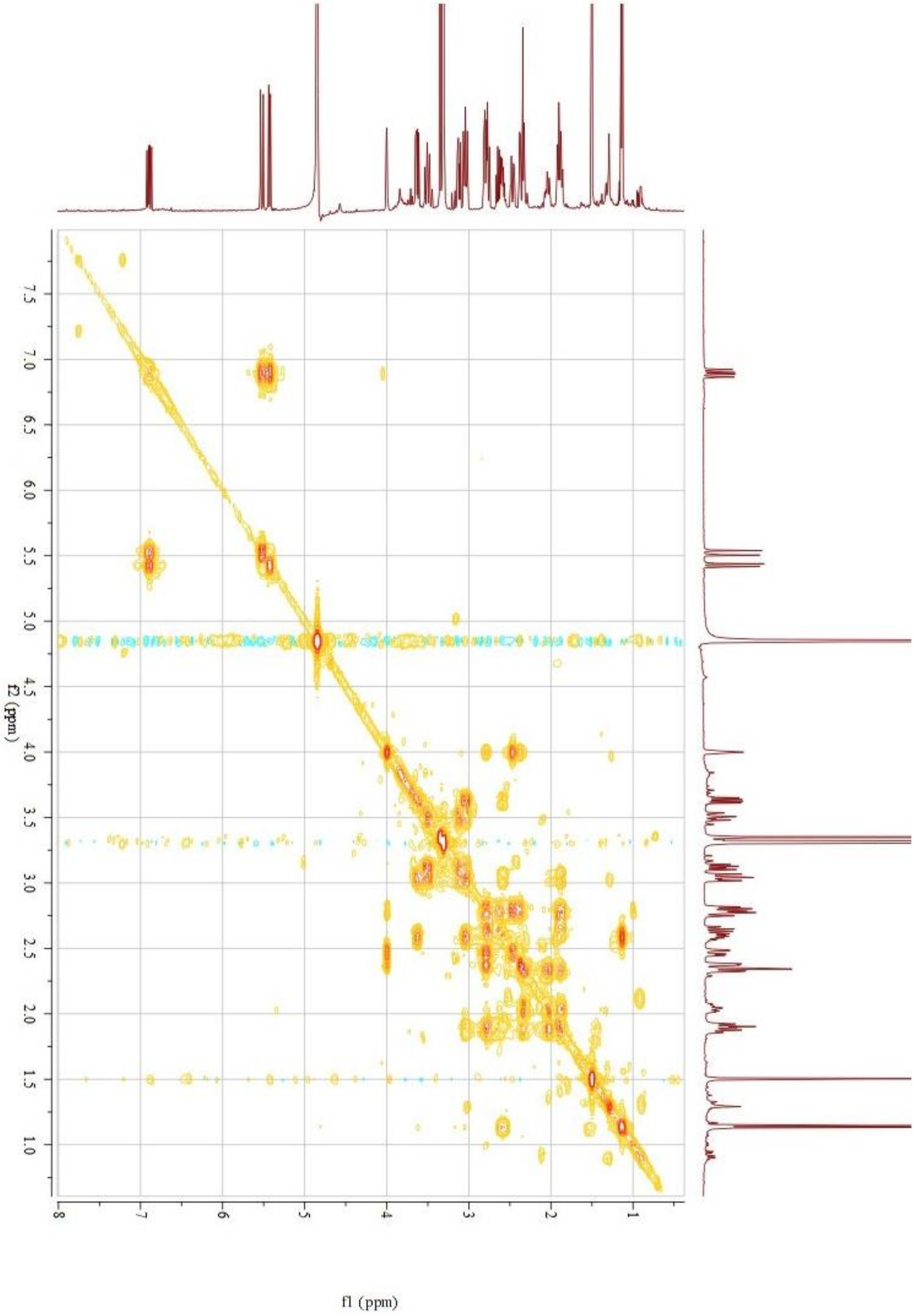

**Figure S32.** HMBC spectrum of calyciphylline R (**4**) in $CD_3OD$.

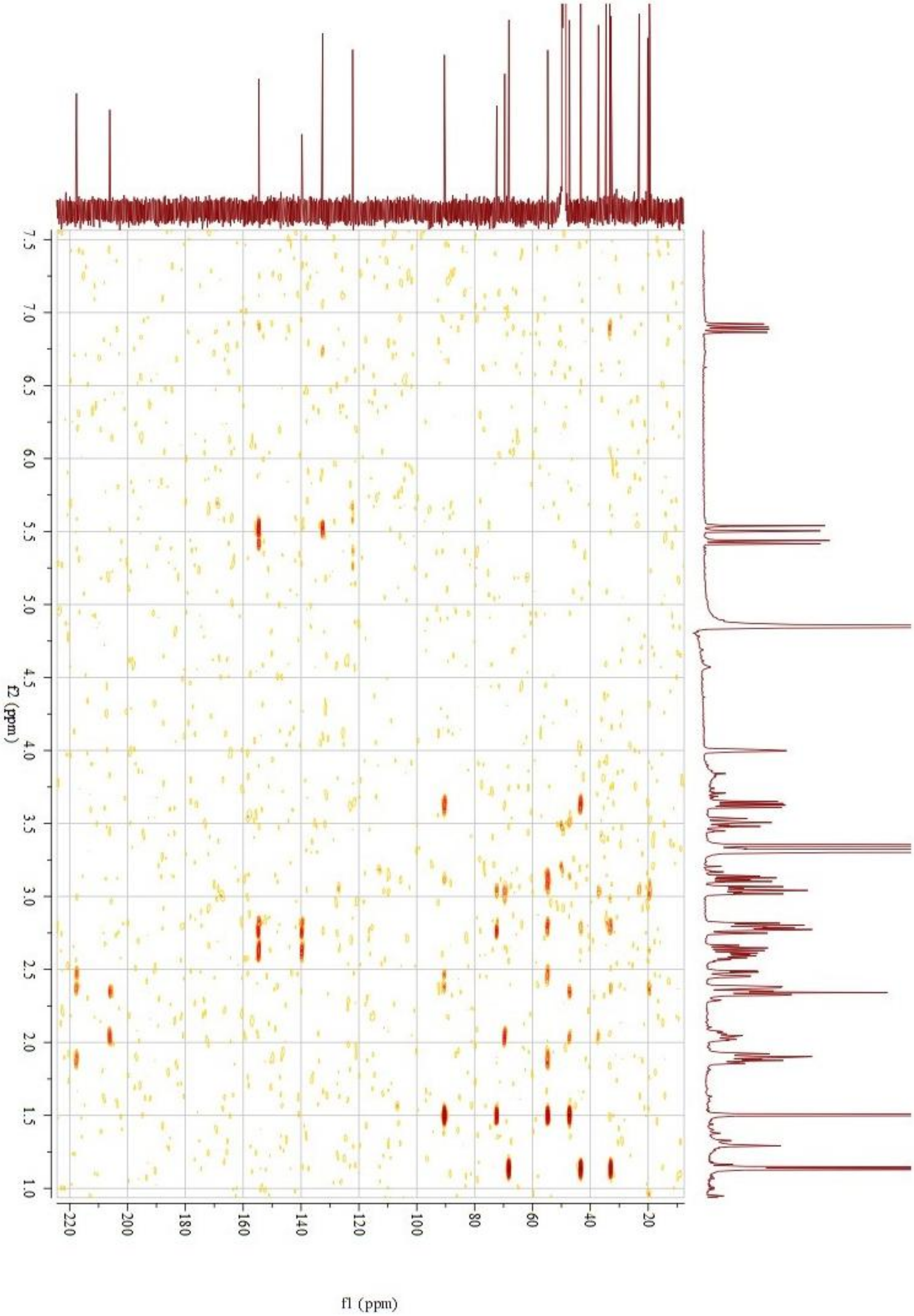

**Figure S33.** NOESY spectrum of calyciphylline R (**4**) in $CD_3OD$.

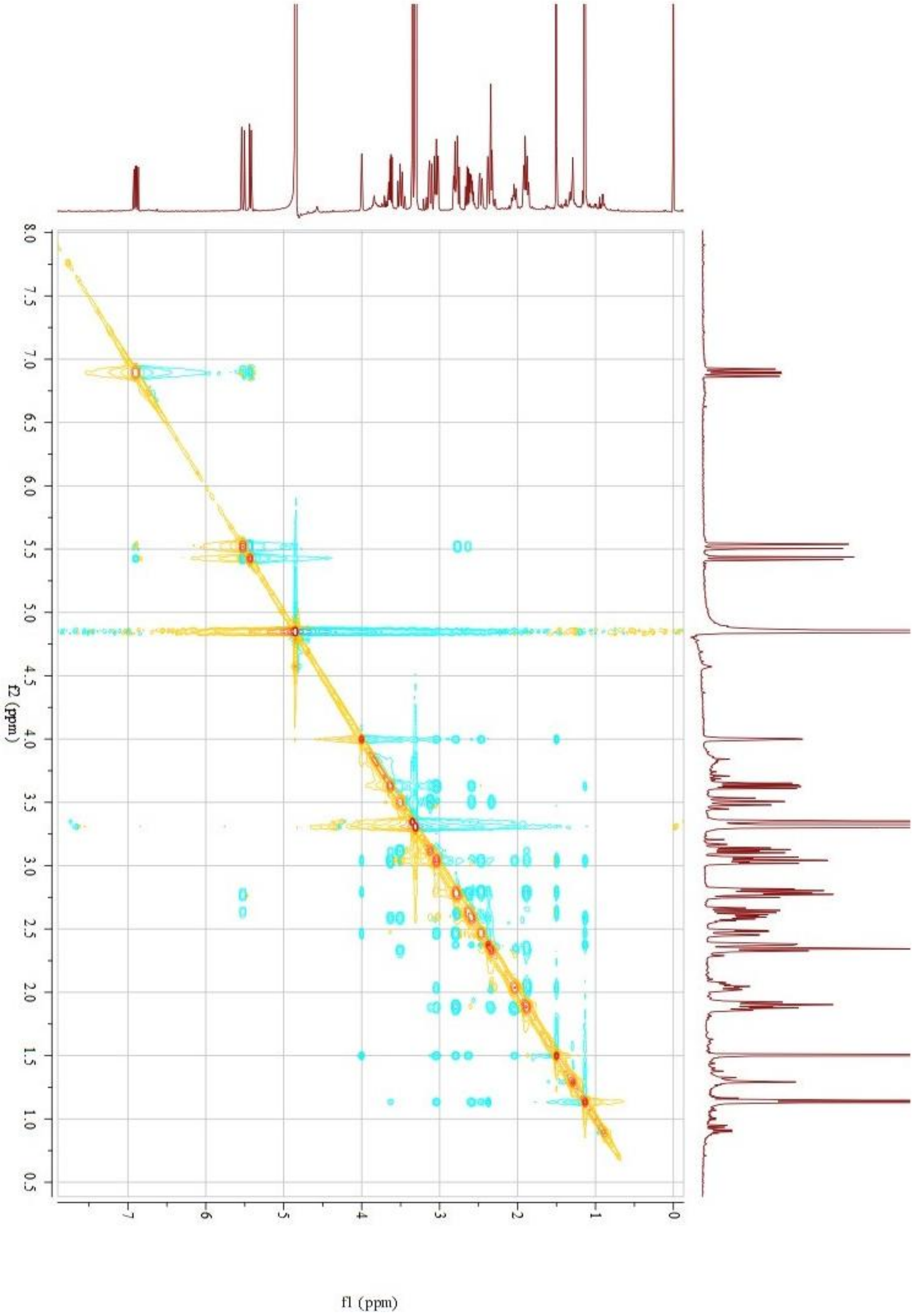

**Figure S34.** ESIMS spectrum of calyciphylline R (**4**).

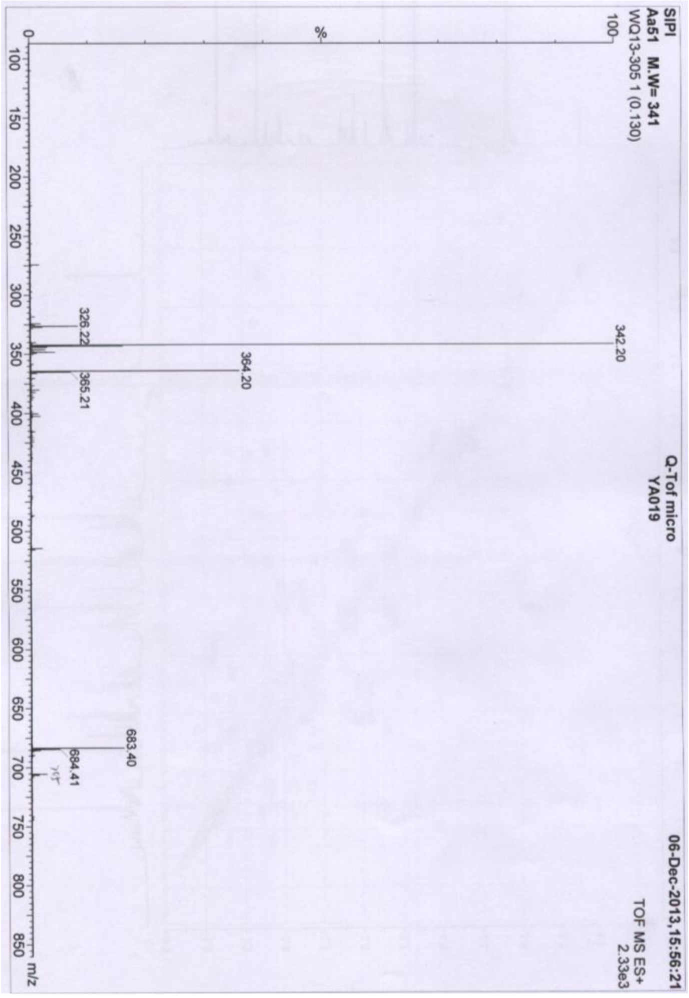


**Figure S35.** HRESIMS spectrum of calyciphylline R (**4**).

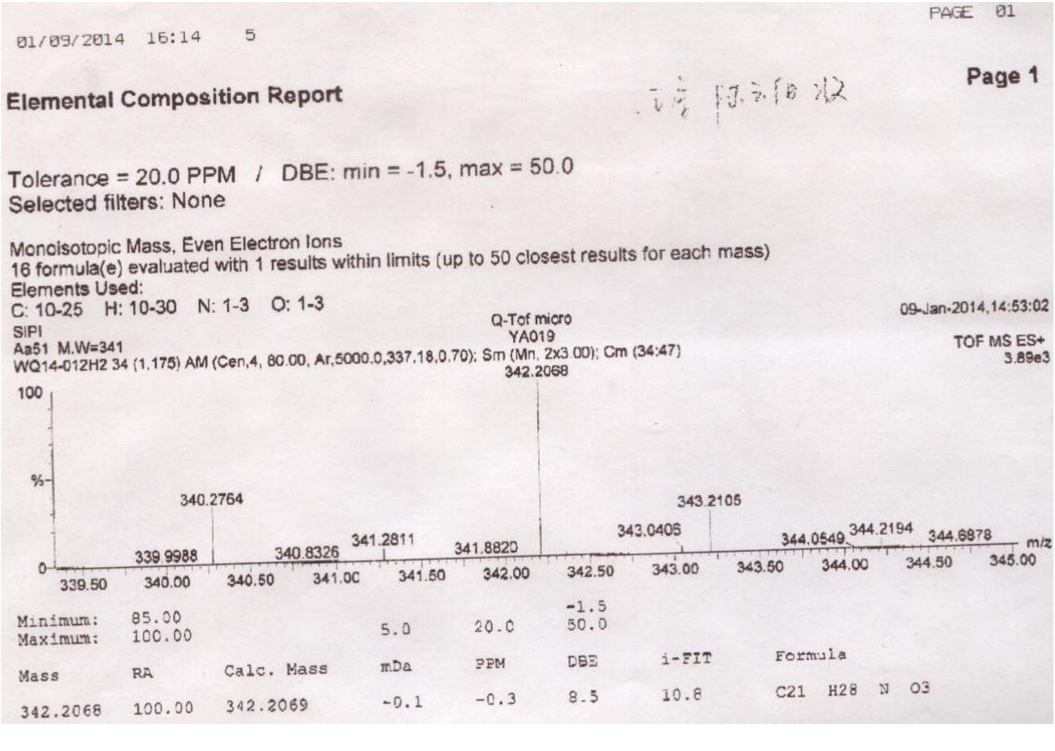
PAGE 01

01/09/2014 16:14 5

Page 1

Elemental Composition Report

Tolerance = 20.0 PPM / DBE: min = -1.5, max = 50.0
Selected filters: None

Monoisotopic Mass, Even Electron Ions
16 formula(e) evaluated with 1 results within limits (up to 50 closest results for each mass)
Elements Used:
C: 10-25 H: 10-30 N: 1-3 O: 1-3



| | | | | | | | |
|---|---|---|---|---|---|---|---|
| Minimum: | 85.00 | | | | -1.5 | | |
| Maximum: | 100.00 | | 5.0 | 20.0 | 50.0 | | |
| Mass | RA | Calc. Mass | mDa | PPM | DBE | i-FIT | Formula |
| 342.2068 | 100.00 | 342.2069 | -0.1 | -0.3 | 8.5 | 10.8 | C21 H28 N O3 |

**Figure S36.** $^{1}$H-NMR (500 MHz, DMSO-$d_6$) spectrum of calyciphylline S (5).

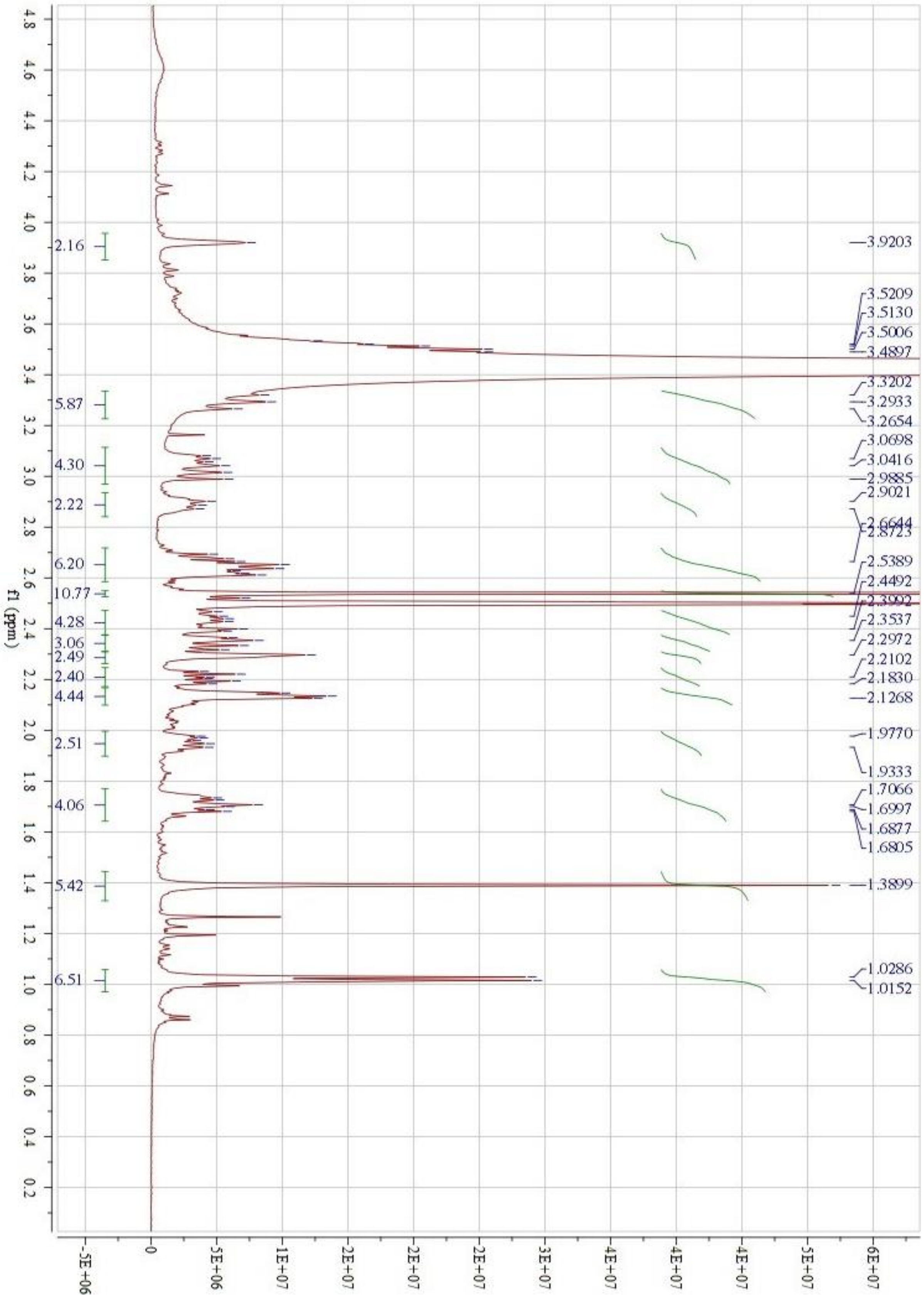

**Figure S37.** $^{13}$C-NMR (125 MHz, DMSO-$d_6$) spectrum of calyciphylline S (**5**).

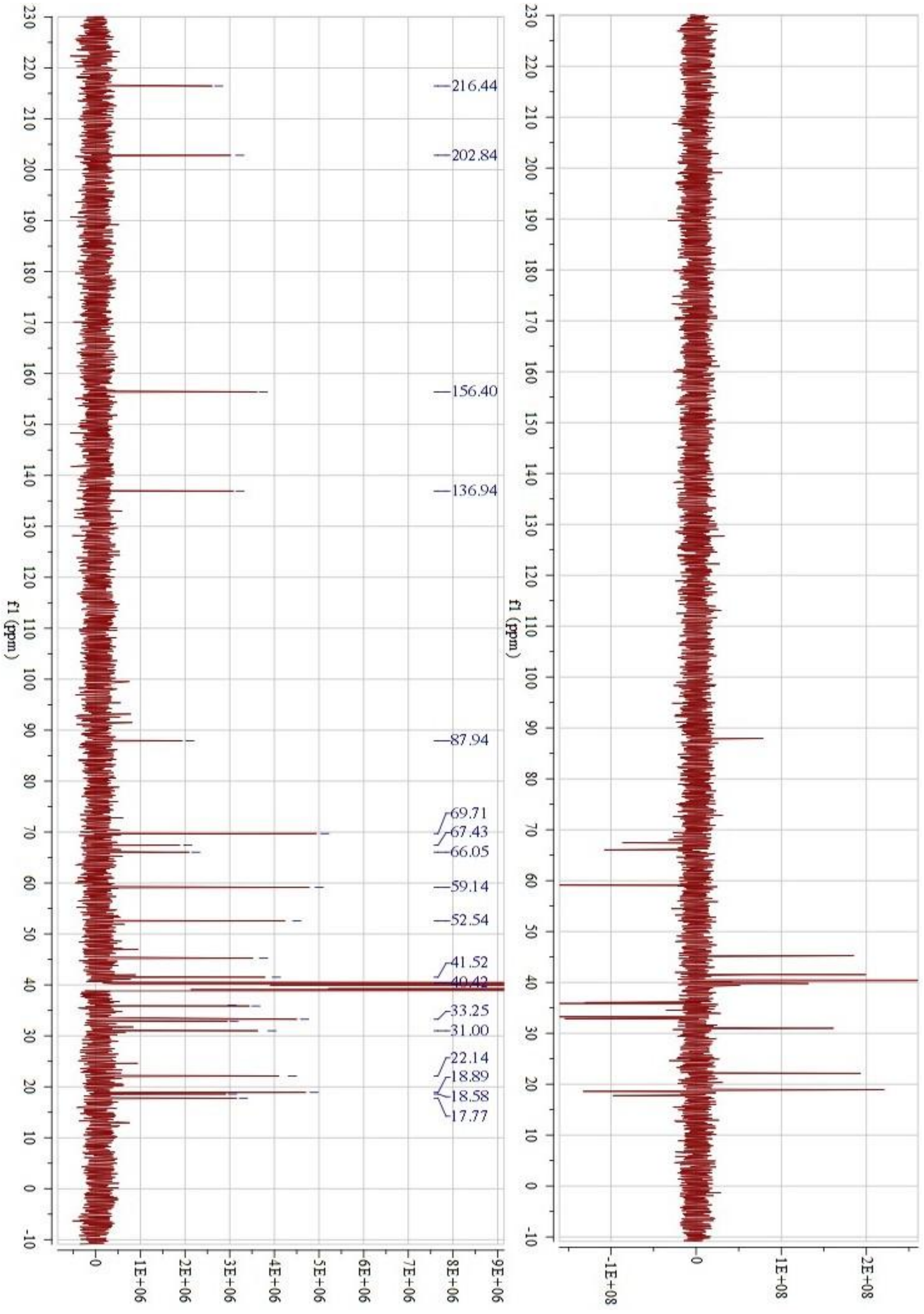

**Figure S38.** HSQC spectrum of calyciphylline S (**5**) in DMSO-$d_6$.

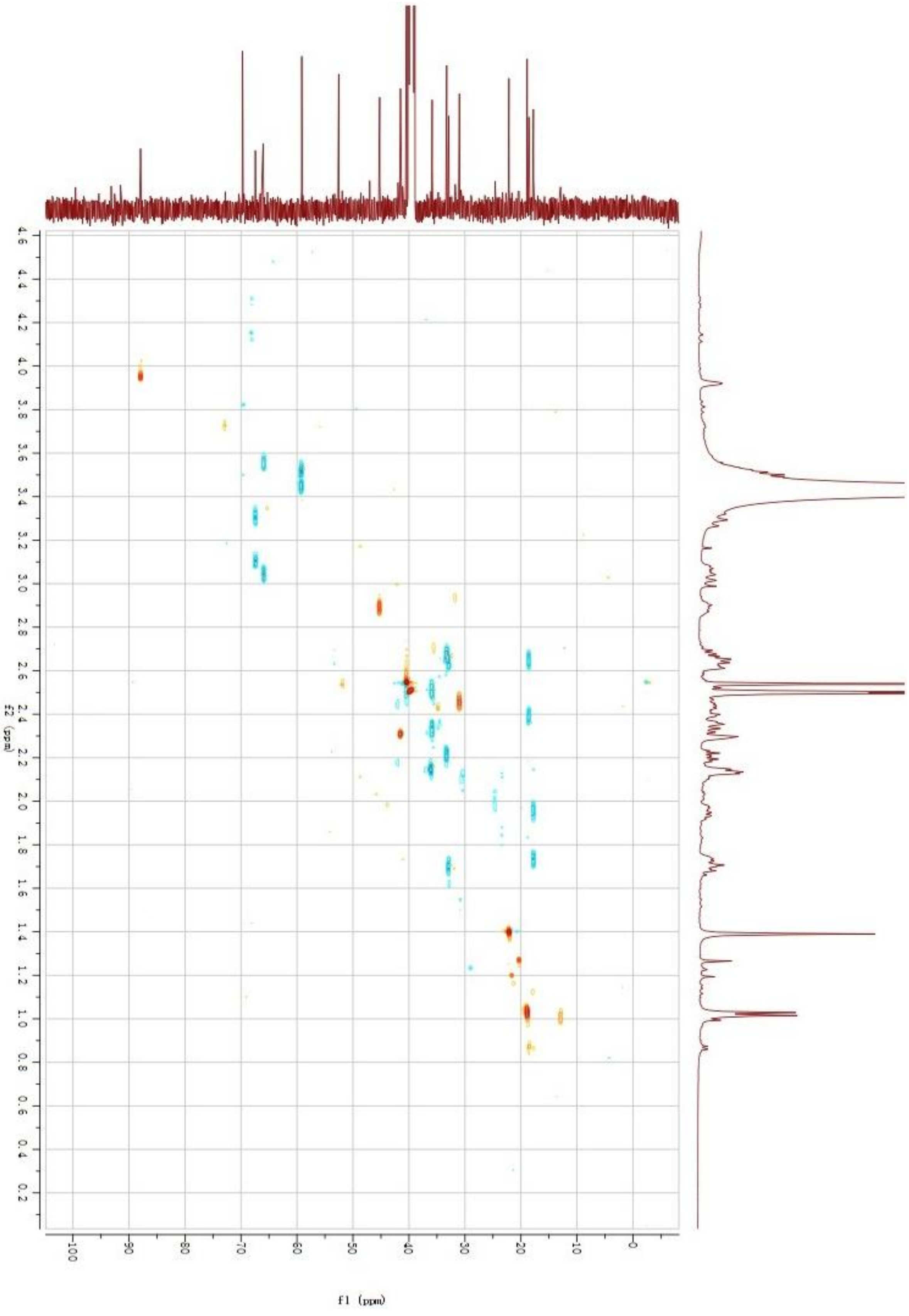

**Figure S39.** $^1H$-$^1H$ COSY spectrum of calyciphylline S (**5**) in DMSO-$d_6$.

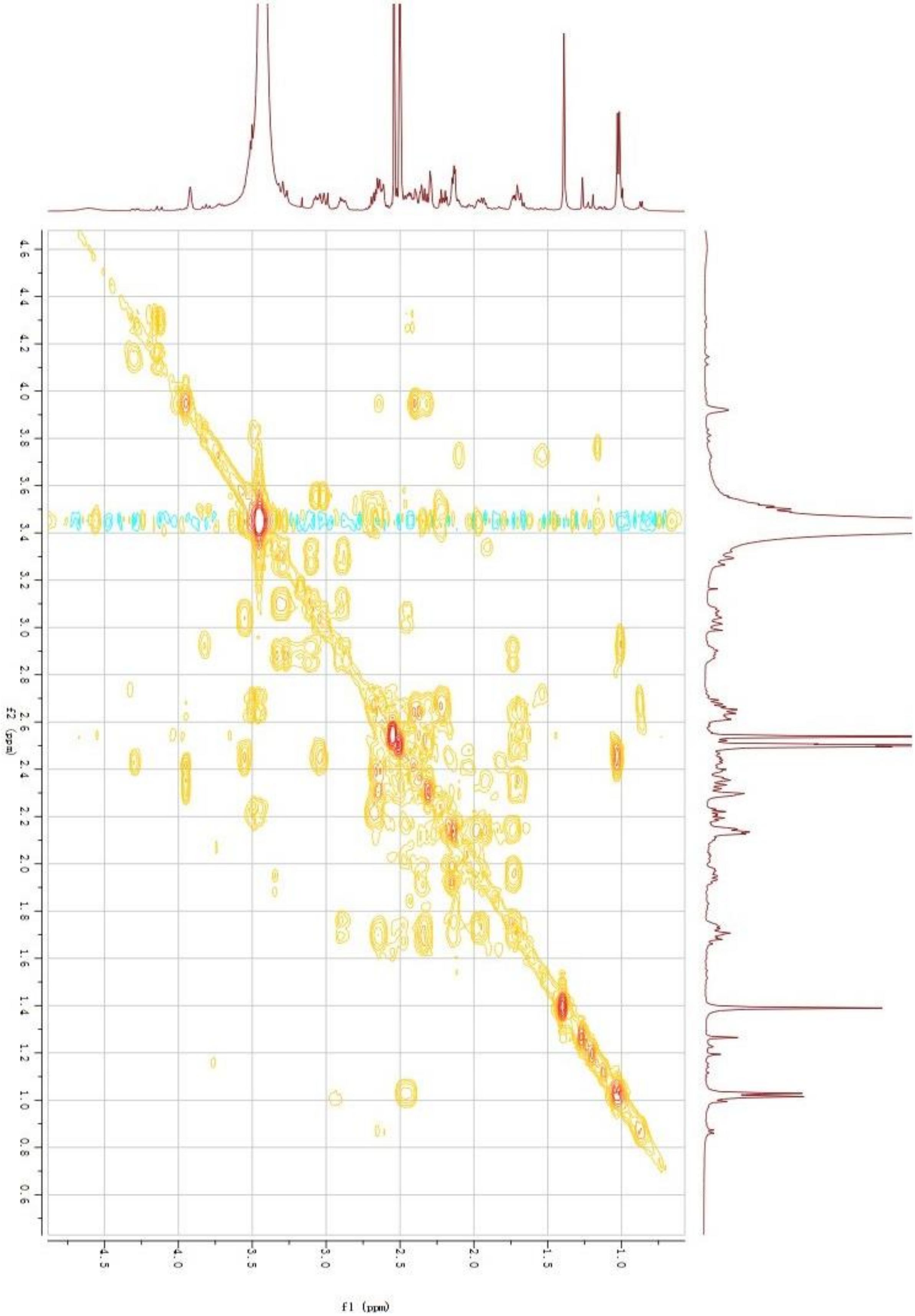

**Figure S40.** HMBC spectrum of calyciphylline S (**5**) in DMSO-$d_6$.

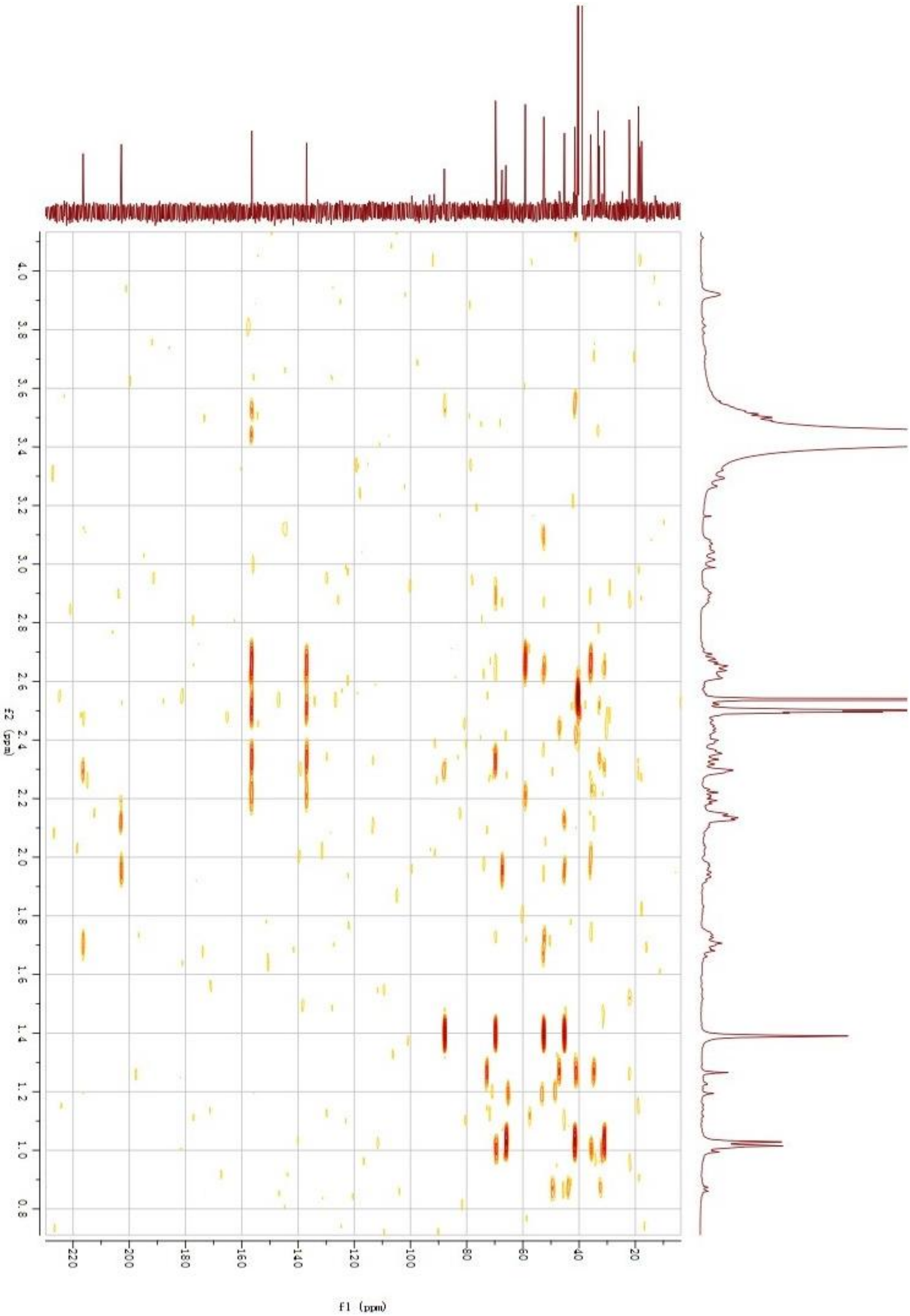

**Figure S41.** ESIMS spectrum of calyciphylline S (**5**).

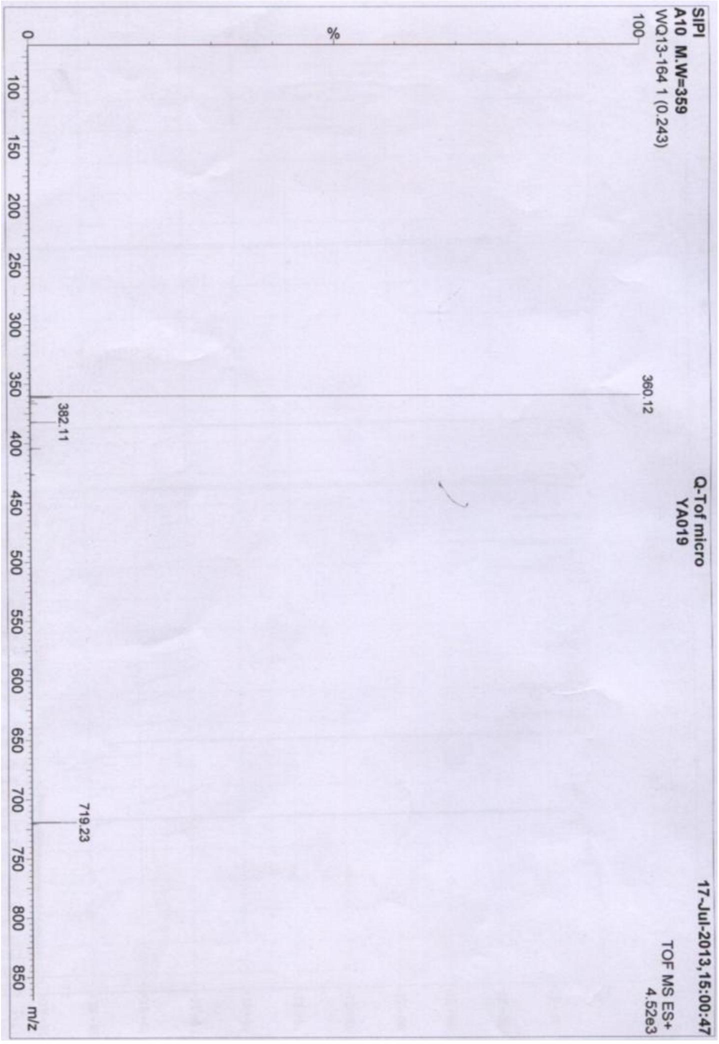


**Figure S42.** HRESIMS spectrum of calyciphylline S (**5**).

**Elemental Composition Report** **Page 1**

Tolerance = 10.0 PPM / DBE: min = -1.5, max = 50.0
Selected filters: None

Monoisotopic Mass, Even Electron Ions
36 formula(e) evaluated with 1 results within limits (up to 50 closest results for each mass)
Elements Used:
C: 5-23 H: 5-35 N: 1-3 O: 1-6

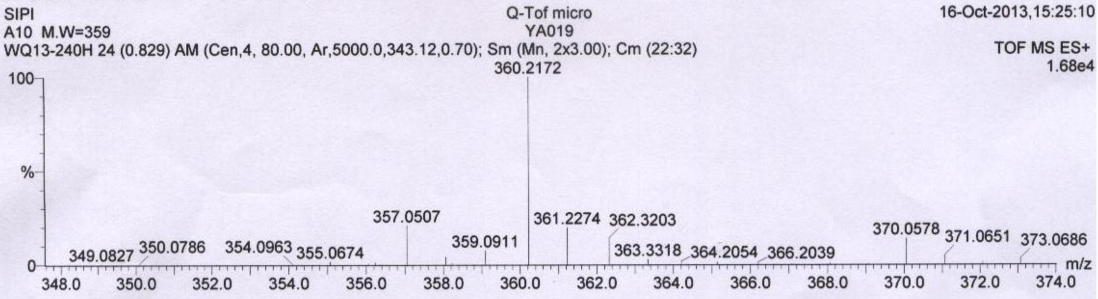


| | | | | | | | |
|---|---|---|---|---|---|---|---|
| Minimum: | 75.00 | | | | -1.5 | | |
| Maximum: | 100.00 | | 5.0 | 10.0 | 50.0 | | |
| Mass | RA | Calc. Mass | mDa | PPM | DBE | i-FIT | Formula |
| 360.2172 | 100.00 | 360.2175 | -0.3 | -0.8 | 7.5 | 2782460.8 | C21 H30 N O4 |

**Figure S43.** $^{1}$H-NMR (500 MHz, $CD_3OD$) spectrum of paxiphylline C (**6**).

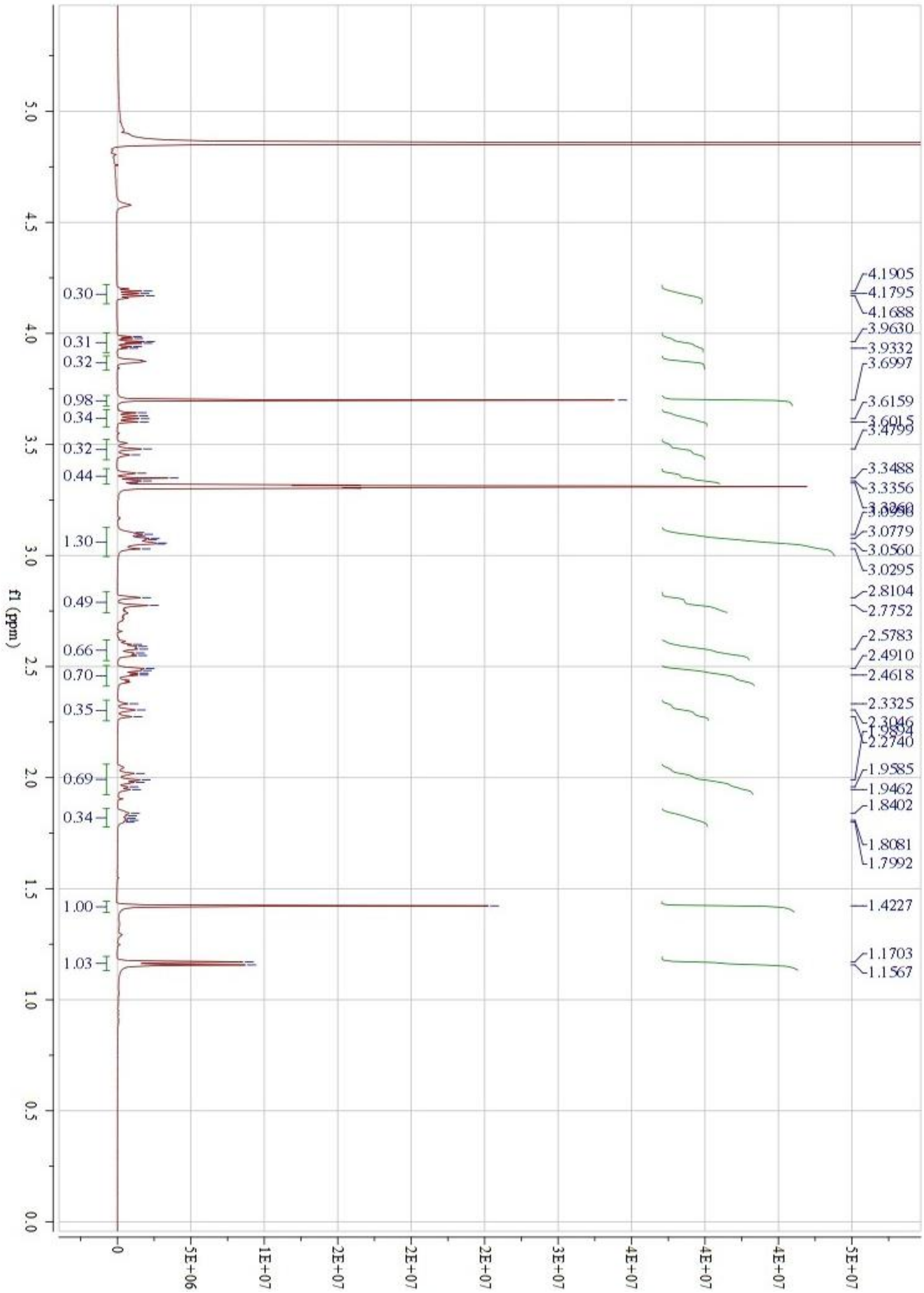

**Figure S44.** $^{13}$C-NMR (125 MHz, $CD_3OD$) spectrum of paxiphylline C (**6**).

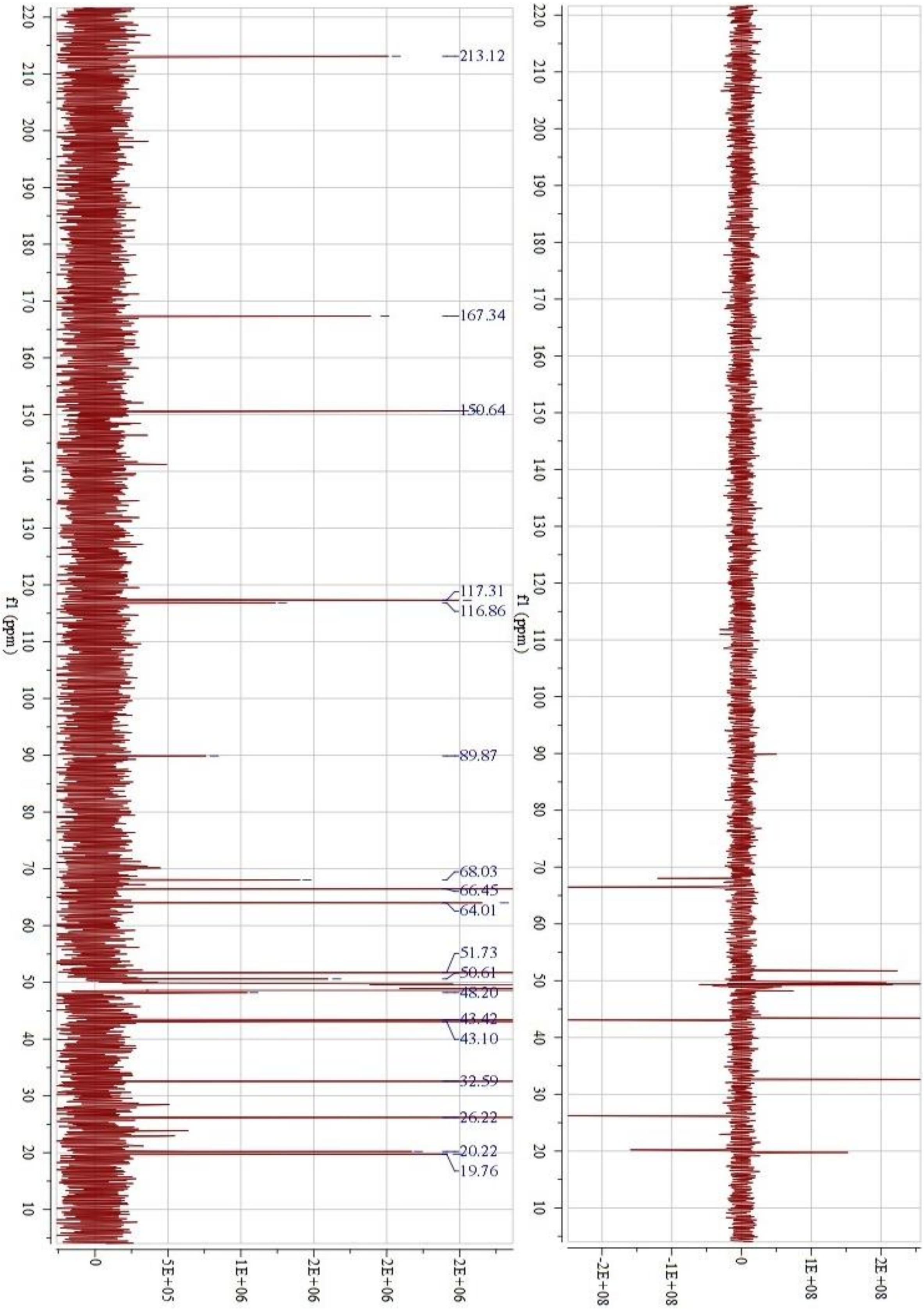

**Figure S45.** $^{1}$H-NMR (500 MHz, DMSO-$d_6$) spectrum of macropodumine B (**7**).

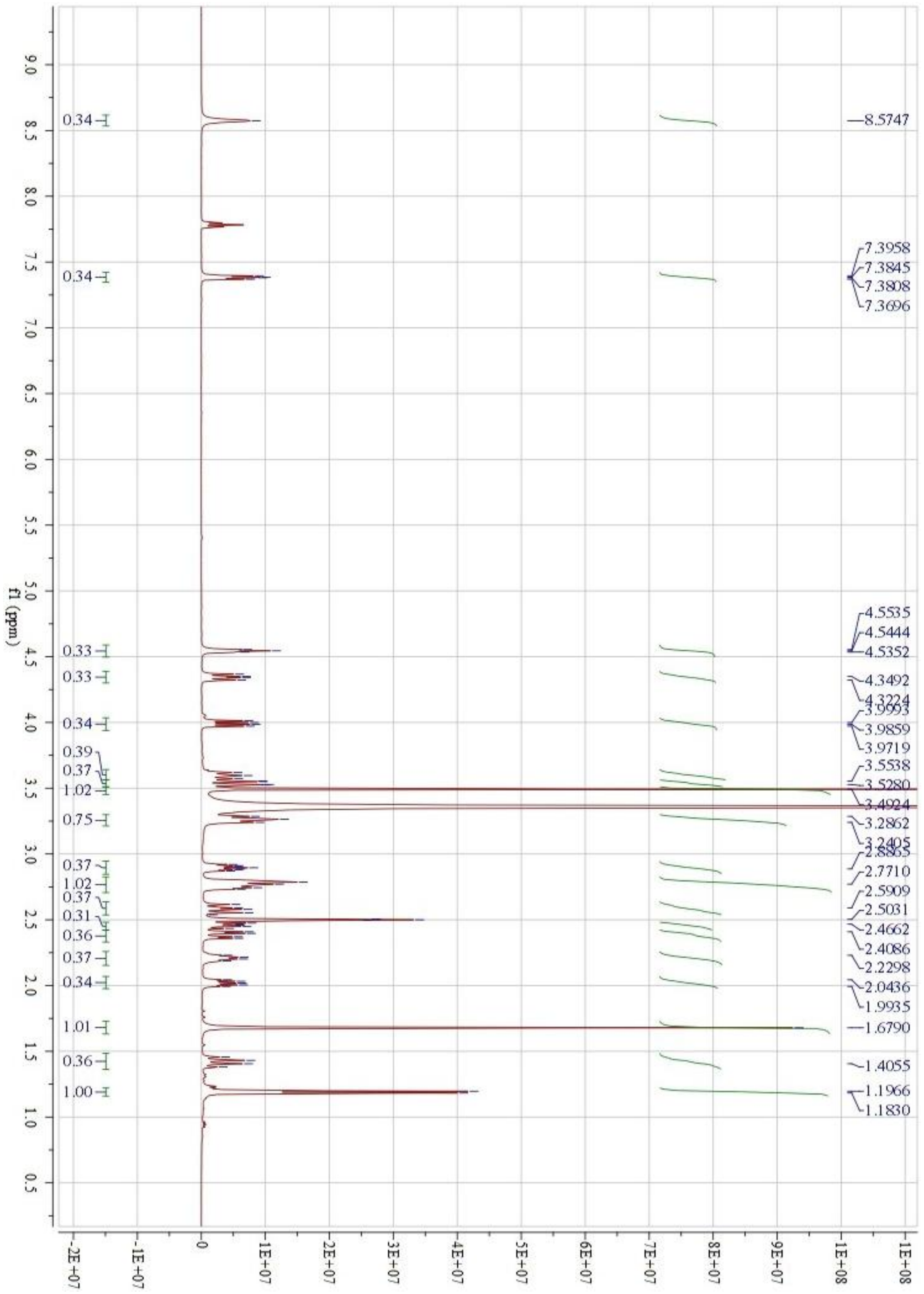

**Figure S46.** $^{13}$C-NMR (125 MHz, DMSO-$d_6$) spectrum of macropodumine B (**7**).

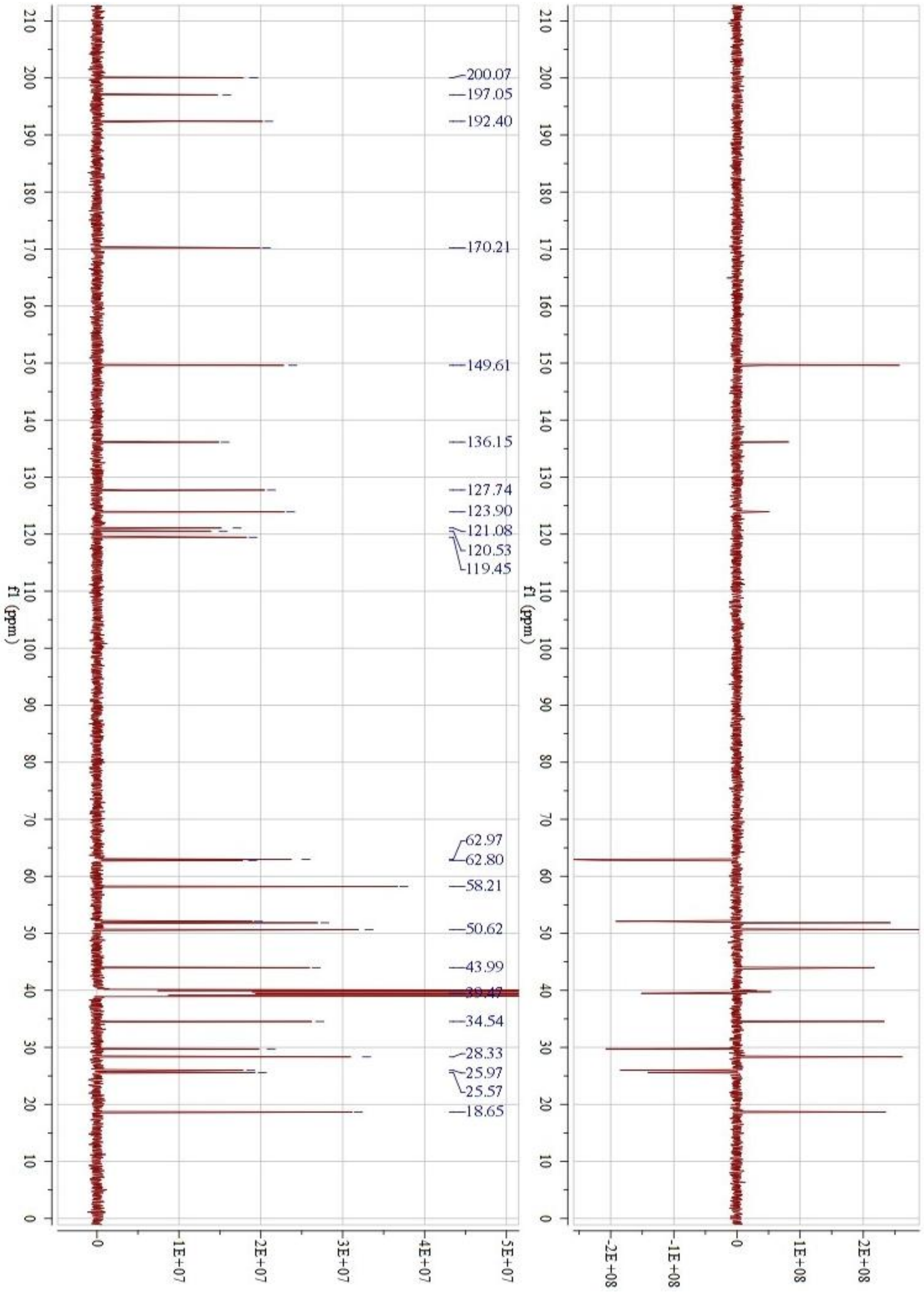

**Figure S47.** $^{1}$H-NMR (500 MHz, $CD_3OD$) spectrum of macropodumine C (**8**).

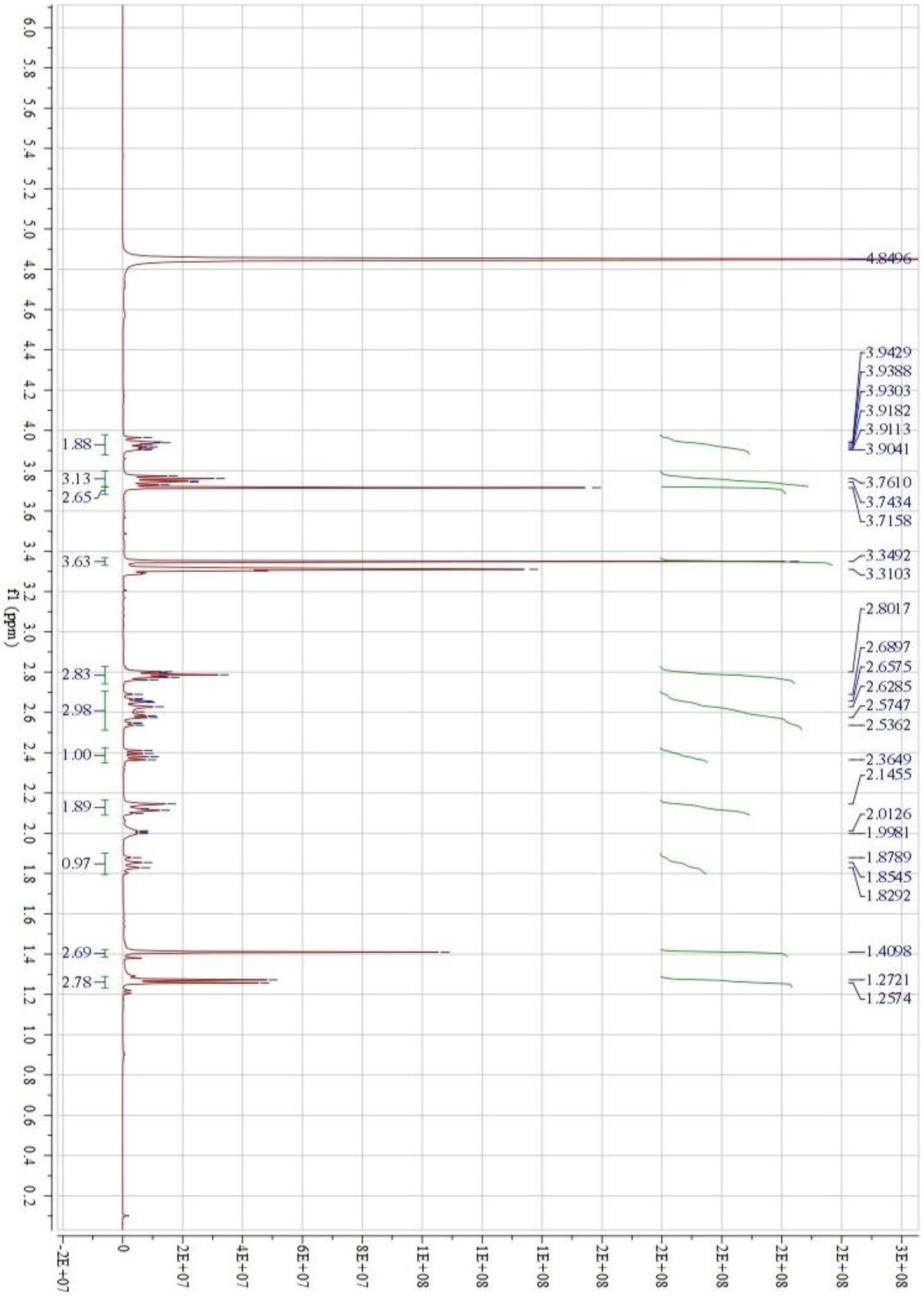

**Figure S48.** $^{13}$C-NMR (125 MHz, $CD_3OD$) spectrum of macropodumine C (**8**).

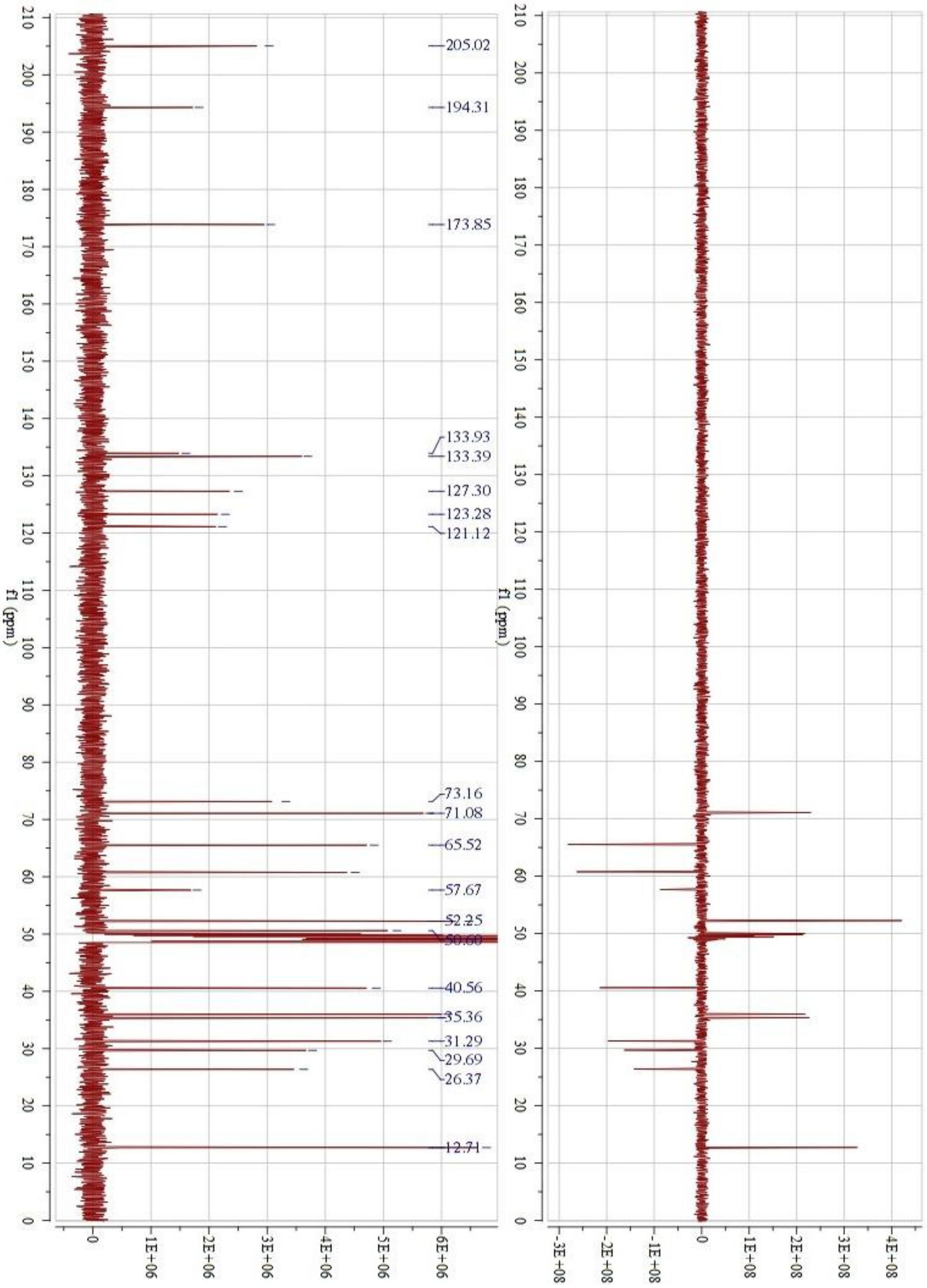

**Figure S49.** $^{1}$H-NMR (500 MHz, DMSO-$d_6$) spectrum of daphnicyclidin A (**9**).

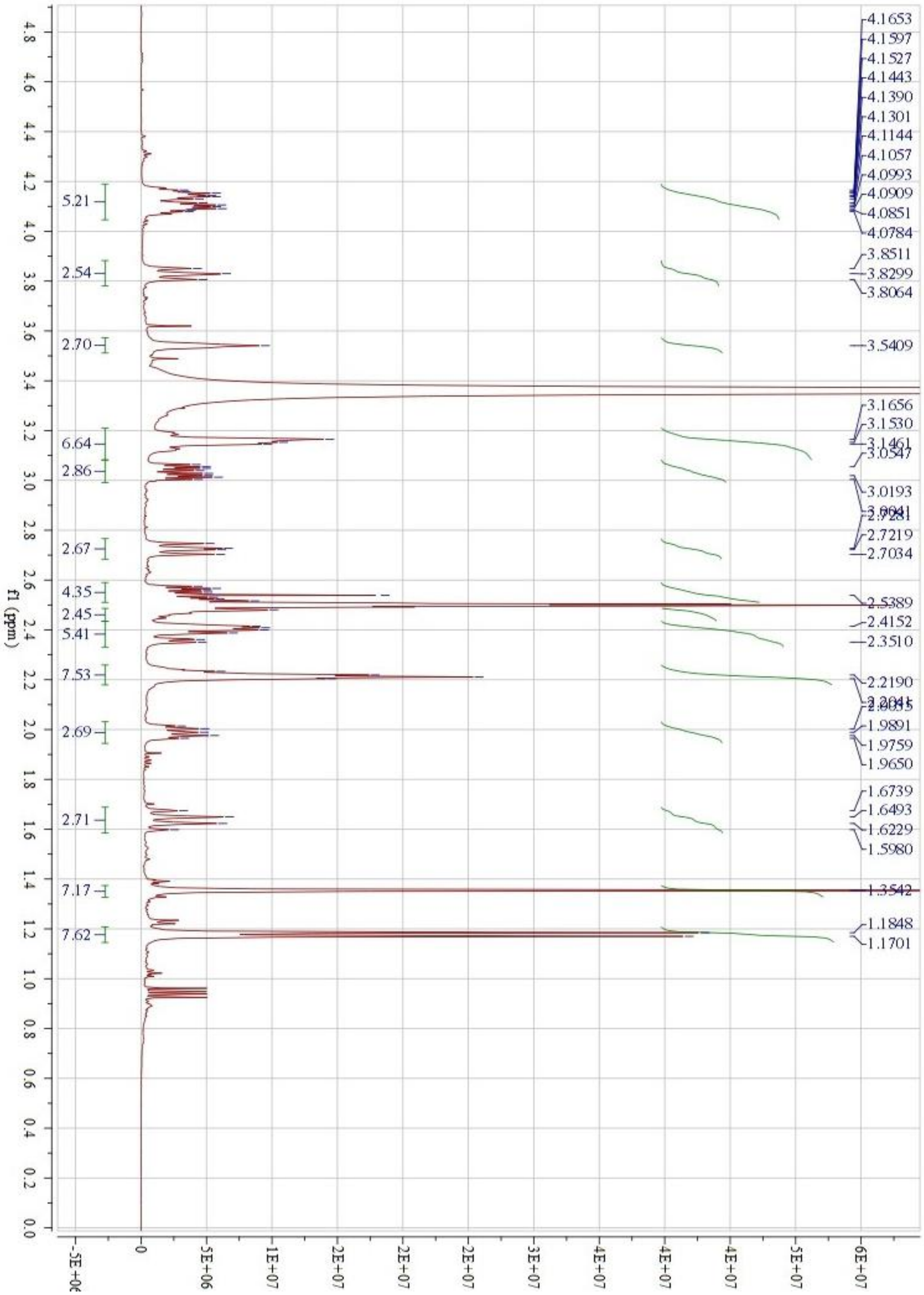

**Figure S50.** $^{13}$C-NMR (125 MHz, DMSO-$d_6$) spectrum of daphnicyclidin A (**9**).

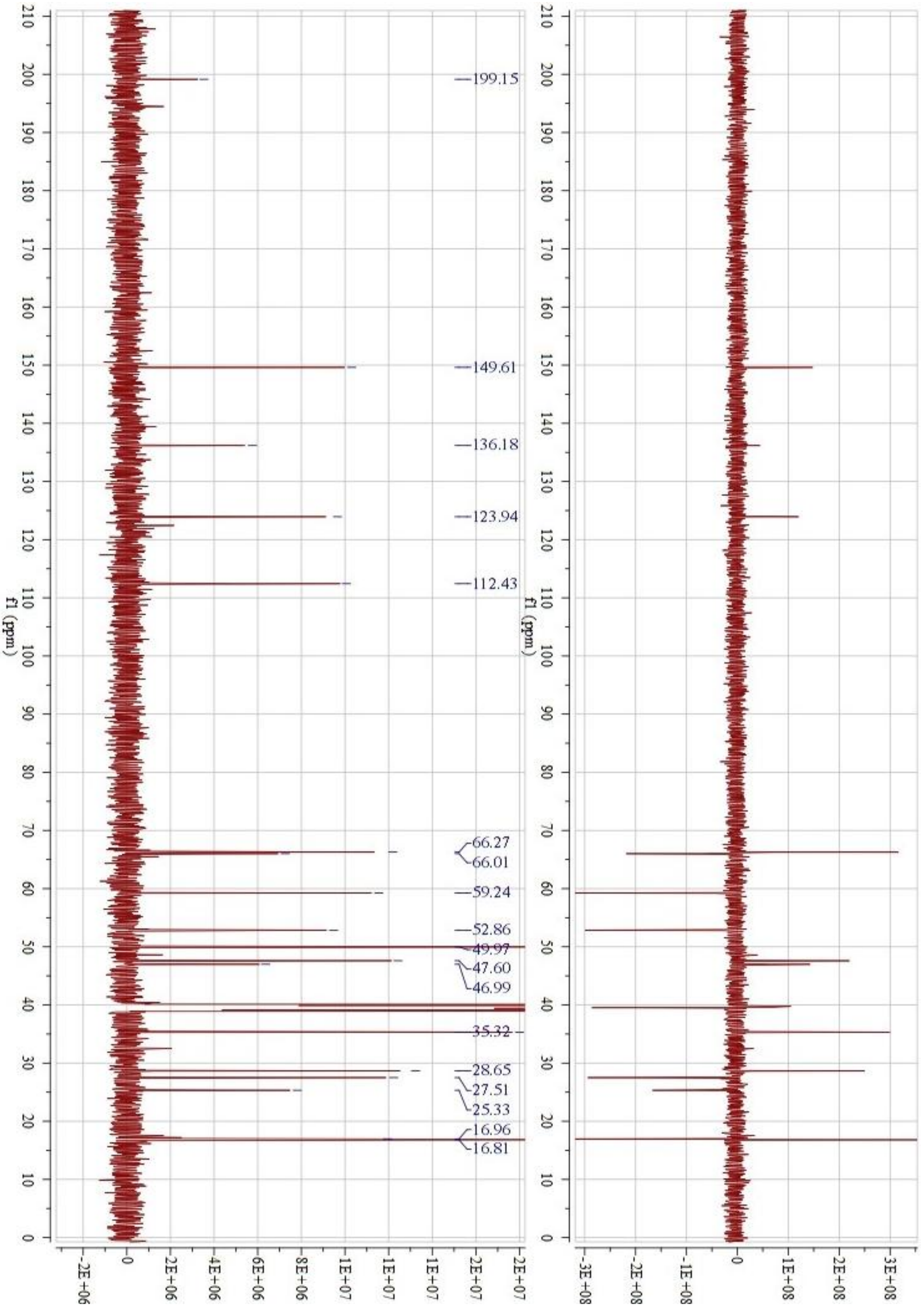

Supplement: Supplementary file 1 [file molecules-19-03055-s001.pdf]
